# CRISPR/Cas9-mediated knockout of factors in non-homologous end joining pathway enhances gene targeting in silkworm cells

**DOI:** 10.1038/srep18103

**Published:** 2015-12-10

**Authors:** Li Zhu, Hiroaki Mon, Jian Xu, Jae Man Lee, Takahiro Kusakabe

**Affiliations:** 1 Laboratory of Insect Genome Science, Kyushu University Graduate School of Bioresource and Bioenvironmental Sciences, Hakozaki 6-10-1, Fukuoka 812-8581, Japan

## Abstract

Gene targeting can be achieved by precise genetic modifications through homology-directed repair (HDR) after DNA breaks introduced by genome editing tools such as CRISPR/Cas9 system. The most common form of HDR is homologous recombination (HR). Binding to the DNA breaks by HR factors is thought to compete with non-homologous end joining (NHEJ), an alternative DNA repair pathway. Here, we knocked out the factors in NHEJ by CRISPR/Cas9 system in silkworm cells, so that increased the activities of HR up to 7-fold. Also efficient HR-mediated genome editing events occurred between the chromosomal *BmTUDOR-SN* gene and donor DNA sequences with an *EGFP* gene in the middle of two homologous arms for the target gene. Utilizing the NHEJ-deficient silkworm cells, we found that homologous arms as short as 100 bp in donor DNA could be designed to perform precise genome editing. These studies should greatly accelerate investigations into genome editing of silkworm.

The silkworm, *Bombyx mori*, is an economic insect and an important model animal for lepidopteron studies. Functional dissection of silkworm genes has been greatly promoted since complement of whole genome sequencing^1^,^2^. Recently, genome editing in silkworm also has been achieved by using DNA nucleases such as zinc finger nucleases (ZFNs)^3^, transcription activator-like effector nucleases (TALENs)^4^,^5^,^6^ and clustered regularly interspaced short-palindromic repeats (CRISPR)-associated Cas9 protein^7^,^8^,^9^. However, investigations on precise genome editing in this kind of insects are still lacking.

Homologous recombination (HR) between transfected DNA and a chromosomal locus can induce precise genome editing, also termed gene targeting. Gene targeting is notoriously difficult in silkworm cells, which do efficiently repair genome DNA by HR only when DNA double-strand breaks (DSBs) are introduced^10^. The nucleases mentioned above (ZFNs, TALENs, and CRISPR/Cas9) has been widely used in various cell types or organisms to bring DSBs in specific genome loci, and repaired by HR pathway with a provided donor DNA to accomplish genome editing. Among these three nucleases, CRISPR/Cas9 nuclease system has been developed rapidly since this technology was used for genome engineering, due to its easy construction and multiplexability^11^. The *Streptococcus pyogenes* Cas9 nuclease is directed by a single guide RNA (gRNA) to 20 bp sequence of target genomic DNA in position next to a 3 bp protospacer adjacent motif (PAM, NGG for SpCas9), generating a blunt-ended DSB. Even that DSBs are introduced in genome, the frequency of HR is inherently low^12^. Once DSB occurs, it will be repaired by several pathways, including HR pathway and non-homologous end-joining (NHEJ) pathway in eukaryotic cells. These two DNA repair pathways always compete with each other for binding to the DSB sites. Inhibition of protein activities in NHEJ pathway such as Ku70, Ku80, Ligase IV (Lig IV) in mammalian cells^13^,^14^ indeed increased the efficiency of precise genome editing mediated by HR pathway. Moreover, the frequency of ZFNs-stimulated HR is increased in Ligase IV-knockout fruit flies^15^,^16^. In silkworm, the frequency of HR is increased in embryos of Ku70 knockout *Bombyx mori*^8^.

To develop a precise and high efficient genome editing method in silkworm cells, here we investigated the frequency of HR for epitope tagging of an endogenous gene, *BmTUDOR-SN*, induced by CRISPR/Cas9 system by using NHEJ-deficient silkworm cells. We found that compared with normal silkworm ovary cells, the cells lacking the key proteins such as Ku70, Ku80, Lig IV, XRCC4 (Lig IV interacting partner), and XLF (XRCC4-like factor) had higher efficiency of homology-directed repair for CRISPR/Cas9-indueced precise gene editing.

## Results

### Construction of a reporter system for detection of NHEJ activity in silkworm cells

**Figure 1:**
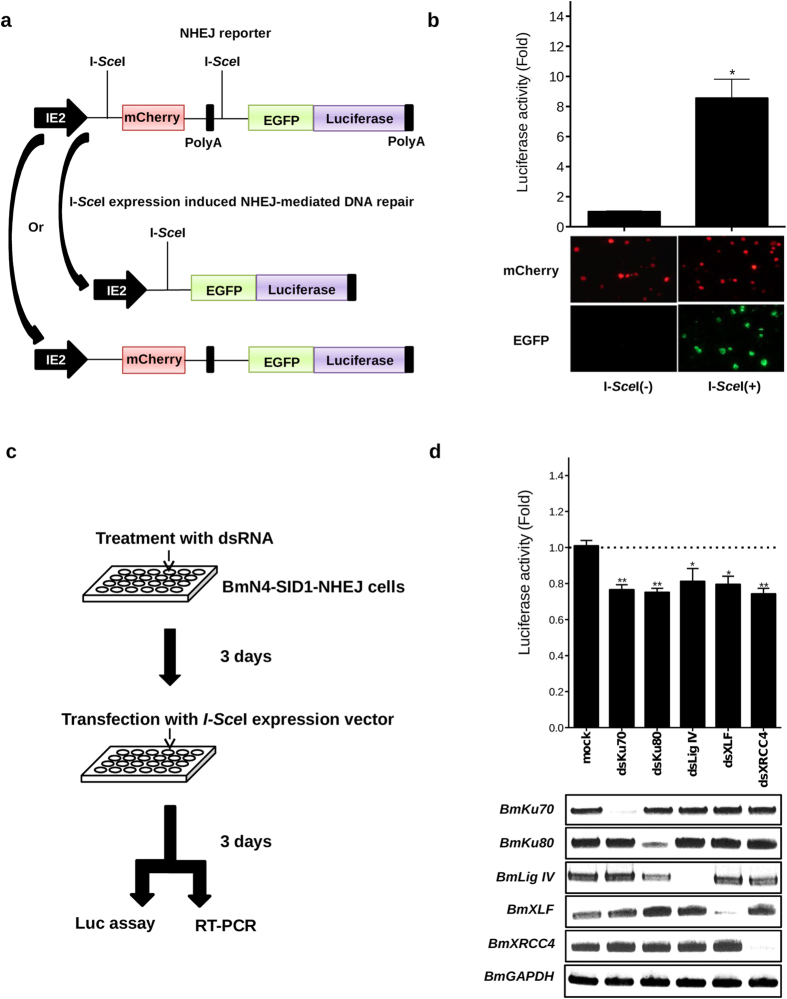
Functional confirmation of NHEJ-related protein genes by a reporter system. (**a**) A NHEJ reporter plasmid was constructed for expression of mCherry under an *IE2* promoter, when no I-*Sce*I expressed in cells. (**b**) The reporter plasmid was co-transfected with helper plasmid into BmN4-SID1 cells, followed by puromycin selection generating NHEJ reporter cells. The cells showed stable mCherry expression. When I-*Sce*I expression vector was transfected into the cells, I-*Sce*I targeting sites were digested. Meanwhile, NHEJ pathway proteins repaired the broken DNA, resulting to construction of a cassette, which expressed EGFP and luciferase proteins. Luciferase assay (Luc assay) was taken after 3 days post-transfection of I-*Sce*I expression vector into NHEJ reporter cells. (**c**) To test the roles of predicted factors involved in silkworm NHEJ pathway, dsRNAs for the factor gens were added into the culture medium of NHEJ reporter cells, followed by transfection of I-*Sce*I expression vector 3 days later. (**d**) Another 3 days later, cells were separated to two parts subjected to Luc assay and RT-PCR, respectively. In (**a**,**d**), the mean fold was derived from values of luciferase assay normalized by the total protein concentrations of cells in each treatment (the control was set as 1 fold indicated by a dotted line). Differences between the means were evaluated with a two-tailed Student *t*-test. Significant differences are as follows: *P < 0.05, **P < 0.01.

We aimed at developing a system of HR-mediated gene targeting with high efficiency, but NHEJ pathway always competed HR for DNA repair at double-strand DNA breaks in eukaryotic cells. In fly and mammalian cells, depletion by RNAi^17^, activity inhibition by a chemical drug^13^,^14^ or knockout of NHEJ-related factors^15^ increased the efficiency of HR-mediated gene editing. In silkworm individuals^8^, knockout of *Ku70* gene could enhance knock-in efficiency. However, it was still unclear which factors were the most critical for NHEJ-pathway mediated DNA repair in silkworm. To analyze the function of NHEJ-related factors, we constructed a reporter system for detection of NHEJ activity in silkworm cells. First, we constructed a vector, which consisted of a mCherry expression cassette bordered by two I-*Sce*I sites and an EGFP-Luciferase coding sequence following the second I-*Sce*I site as depicted in Fig. 1a. Then, the vector was integrated into genome of silkworm BmN4-SID1 cells with a *piggybac* transposable element as described previously^18^. After puromycin selection, the transgenic cells, named BmN4-SID1-NHEJ, expressed mCherry stably, as shown in Fig. 1b. When an I-*Sce*I expression vector was transiently transfected into the cell line, we found clear EGFP signals and strong luciferase activity in the cells after 5 days post transfection (Fig. 1b), indicating NHEJ pathway occurred and repaired genome DNA by ligation of the two sites cut by *I-Sce*I. Because there must be perfect DNA repair producing the DNA structure as the original sequence (Fig. 1a) in the cells and some cells was not transfected with I-*Sce*I expression vector, thereby showing the mCherry signals only (Fig. 1b). Using the BmN4-SID1-NHEJ cells, we knocked down the putative NHEJ-related factor genes nas the procedure illustrated in Fig. 1c. Luciferase assay (Luc assay) data (Fig. 1d) indicated that knockdown of NHEJ-related factor genes (*BmKU70*, *BmKU80*, *BmLIG IV*, *BmXLF*, and *BmXRCC4*) affected the activities of NHEJ for DNA repair. Semi-quantified RT-PCR (below the Luc assay data, Fig. 1d) was performed to detect the mRNAs of NHEJ-related genes were knocked down after dsRNA treatments.

### Using an All-In-One CRISPR/Cas9 expression vector to knock out NHEJ-related genes in silkworm cells

**Figure 2:**
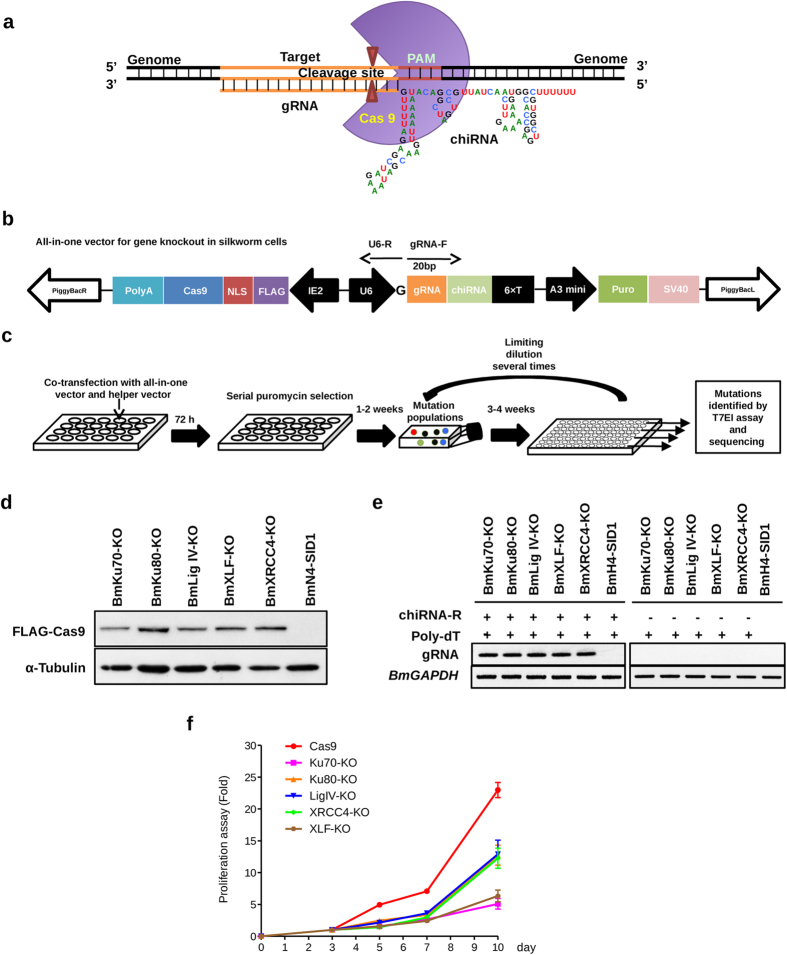
An “All-In-One” vector was constructed for CRISPR/Cas9-mediated knock-out silkworm cells. (**a**) A schematic representation of Cas9-gRNA complex used in this study. (**b**) A schematic representation of “All-In-One” vector. The plasmid consists of three cassettes sandwiched by inverted terminal repeat sequences of *piggyBac* transposon system for transgenic expression of Cas9, gRNA, and puromycin, independently. Cas9 coding sequence was fused with a polyA signal, a nuclear localization signal (NLS), and a 3 × FLAG tag under an IE2 promoter^26^. gRNA expression was controlled by a silkworm U6 promoter^27^ with a transcription stop signal (6 × T). Puromycin expression was under the control of actin A3 promoter^10^ with a transcription stop signal (SV40). (**c**) After co-transfection with the All-In-One vectors targeting to NHEJ-related protein genes and a helper plasmid into BmN4-SID1 cells, a series of puromycin selection was performed. About 1–2 weeks later, a stable cell line containing various mutant cells and normal cells was obtained. With limiting dilutions by 96-well plates several times (diluted as 10 cells/well every time), mutant cells were enriched. Mutations were confirmed by T7 endonuclease I (T7EI) assays and sequence analysis. (**d**) After enrichment of NHEJ-related gene mutant cells, the cell lysates were subjected to western blot, with FLAG antibody (1:3000) and α-Tubulin antibody (1:1000), separately. (**e**) RNA was extracted from the mutant cells, followed by RT-PCR to check the expression of gRNA. The primer (chRNA-R) was added (+) or not (−) to do reverse transcription. (**f**) Knockout of NHEJ-related genes affected the proliferation of silkworm cells. The numbers in the y-axis represent fold changes derived from the OD values with measuring the absorbance at 450 nm using a microplate reader. Data are expressed as the mean ± SEM from three independent experiments.

An All-In-One vector (Fig. 2b) was constructed as described in Methods for expression of Cas9 and gRNA, simultaneously. The All-In-One vector contained a puromycin expression cassette for selection of positive cells expressing Cas9 and gRNA. To construct NHEJ-related gene knockout cells, gRNAs targeting NHEJ-related genes were designed by searching 19 or 20 nt target sequence upstream of the PAM (NGG). The primers (Table S2) containing gRNA and All-In-One vector sequences were used to make insertion of the gRNA expression region in the All-In-One vector by a PCR-mediated mutagenesis (details in Methods). Due to the U6 promoter for gRNA expression, all gRNAs were added a G as the first base. We did not comply with the rule as G-(N)_20_-NGG to search gRNA targets, so that some gRNAs with an appended G would not perfectly match target sequences as shown in supplementary Table S2. After co-transfection of the All-In-One vector with *piggyBac* helper transposase expression vector into BmN4-SID1 cells, a series of puromycin selection and limiting dilution was taken as illustrated in Fig. 2c. About one mouth later, stable cell lines in puromycin-containing medium were obtained. Western blot was performed to detect Cas9 expression in the cell lines (Fig. 2d). gRNA expression was confirmed by RT-PCR with Poly-dT as the primer for reverse transcription (Fig. 2e). We also investigated the cell growth by a cell counting kit (CCK-8). As shown in Fig. 2f, compared with Cas9-only expression cells, all the cells deleted NHEJ-related genes grew slowly. Because of the low growth rate, we found single cell per well in 96-well plates grew very slowly and many cells died after dilution in 96-well plates. Finally, we found 10 cells per well in 96-well plates grew well and finally obtained colonies after a series of limiting dilutions.

**Figure 3:**
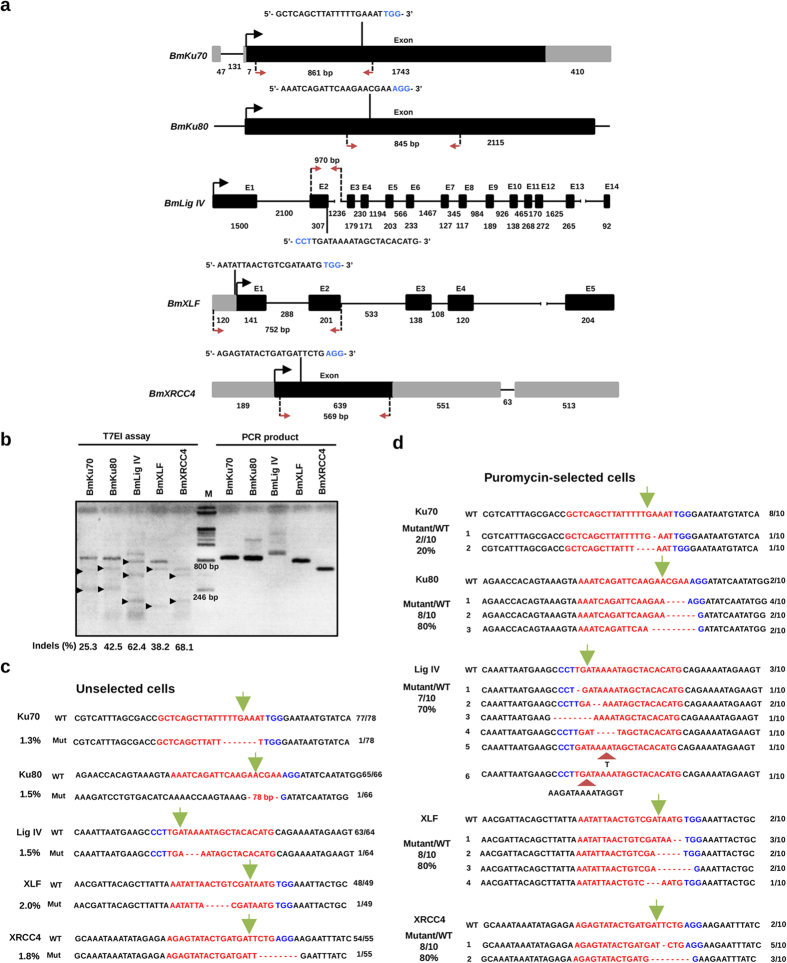
CRISPR/Cas9-mediated NHEJ-related gene mutations in silkworm cells. (**a**) Design of gRNAs targeting to NHEJ-related genes. Designed gRNAs were marked on the top of the gene structures. Nucleic acids marked by blue were Proto-spacer Adjacent Motifs (PAM). Gray boxes and black boxes represent untranslated regions and depicting exons, respectively. Black lines between exons represent introns. Black arrows indicate the starting point for translation. Red arrows indicate the targeted regions, which were cloned for sequence analysis. (**b**) T7EI assay was performed to detect the mutants. Black arrows indicate the predicted sizes of T7EI digestion. PCR products amplified form the target regions were used as negative controls. Primers used for the PCR reactions are listed in Table S5, and the percentage of cleaved band was measured using Image J software. The result was representative of three independent experiments. Numbers below the bands indicate average percentage of Indels (n = 3). (**c**) Sequences of gRNA target sites in the puromycin-unselected cells. BmN4-SID1 cells was transiently transfected with the All-In-One vectors. One week later, the cells were used for genome extraction, with which the target regions were amplified and cloned for sequence analysis. Red color marked nucleic acids indicate gRNA target sequences, while the blue ones indicate PAMs. Deletions were modified by dashed lines. Green arrows pointed to the sites cut by Cas9 protein. The fractions listed on the right indicate the numbers of mutations or wild type in the total sequenced clones. The numbers of percentage on the left indicate the mutation rates in the sequenced colonies. (**d**) Sequences of gRNA target sites in the puromycin-selected cells. Signs were used as the same as in C. Besides, insertions were marked with red arrowheads.

To analyze the mutagenesis mediated by CRISPR/Cas9 in the silkworm BmN4-SID1 cells, T7 endonuclease I (T7EI) assay was performed using the PCR products amplified from the genomic DNA of the transgenic cells after puromycin selection. T7EI assay indicated that Cas9 were guided by gRNA to the target region of genome as depicted in Fig. 3a, and successfully cut the target DNA into expected sizes (Fig. 3b). In addition, the LigIV lane shows 3 obvious bands this time, suggesting that different type of mutants exist in the cell population. To confirm T7EI assay results, we cloned the PCR fragments amplified from the puromycin-selected and none puromycin-selected cell genomic DNAs and sequenced many bacterial colonies. The data (Fig. 3c,d) indicated that mutations occurred in the genome of cells after transfection with All-In-One vectors, and puromycin selection could greatly increase the mutation rates in the genes such as *BmKU70* from 1.3% to 20%, *BmKU80* from 1.5% to 80%, *BmLIG IV* from 1.5% to 70%, *BmXLF* from 2.0% to 80%, *BmXRCC4* gene from 1.8% to 80%.

### Increased HR efficiency in the NHEJ-deficient silkworm cells

**Figure 4:**
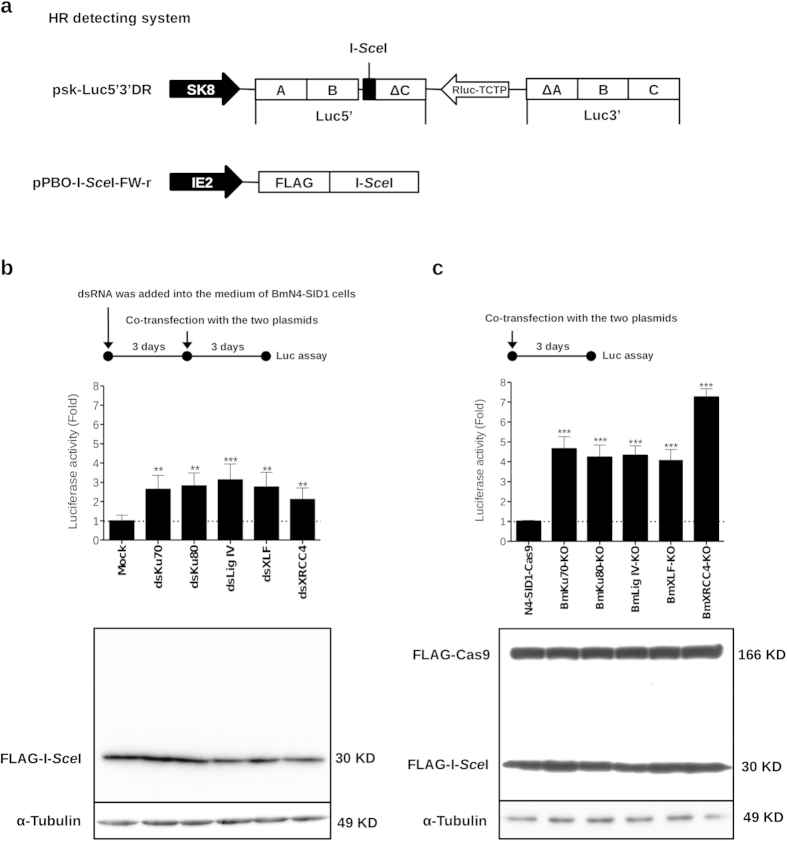
Knockdown and knockout of NHEJ-related genes increased the HR activity in silkworm cells. (**a**) A schematic representation of plasmids for HR reporter system. This system was constructed before^18^. (**b**) BmN4-SID1 cells were treated with dsRNAs against NHEJ-related genes for 3 days, followed by co-transfection with pSK8Luc5′3′DR and pPBO-I-*Sce*I-FW-r. Another 3 days later, cells in each well were separated into two parts for Luc assay and western blot, respectively. Cells treated without dsRNA (Mock) was used as a negative control. (**c**) Mutant cells were prepared in 24-well plates at the density of 1 × 10^5^ cells/well for 16 h, followed by co-transfection with the reporter plasmids. Luc assay and western blot were performed after 3 days later. The cell line named N4-SID1-Cas9, which transgenically expressed Cas9 protein in BmN4-SID1 cells, was used as a negative control. In B and C, the mean fold (the controls were set as 1 fold indicated by dotted lines) was derived from values of luciferase assay normalized by the total protein concentrations of cells in each treatment. Differences between the means were evaluated with a two-tailed Student *t*-test. Significant differences are as follows: **P < 0.01, ***P < 0.001. The western blot data was a representative from repeated three independent experiments.

Previously, we developed a luciferase-based assay system for the analysis of HR activity^18^ in silkworm cells. In this system (Fig. 4a), one plasmid named psk-Luc5′3′DR, which contained a nonfunctional *luciferase* gene with an I-*Sce*I site, and an I-*Sce*I expression vector. When I-*Sce*I induced a DSB within the nonfunctional *luciferase* gene, efficient HR would repair the DSB by gene conversion using the Luc3′ region as depicted in Fig. 4a. To analyze the effect of knockdown of NHEJ-related gene on HR efficiency, we treated BmN4-SID1 cells with dsRNAs against the NHEJ-related genes, followed by transfection with the plasmids in the HR detecting system. The results of Luc assay (Fig. 4b) indicated that depletion of NHEJ-related genes increased HR efficiency. By taking the same system, we also detected HR efficiency in the NHEJ-related gene knockout cells. As shown in Fig. 4c, HR activities in all knockout cells were higher than the control cell line, which expressed Cas9 protein only. To exclude the possibility that I-*Sce*I protein, the DSB trigger, was not consistent in the control cells, dsRNA-treated cells, or gene knockout cells, we performed western blot, which showed the same protein levels of I-*Sce*I and Cas9 proteins in the cell lysates used for Luc assay. Moreover, it was notably that HR efficiency was greatly increased and higher in the knockout cells than dsRNA treated cells. For example, BmXRCC4-KO cells (Fig. 4c) showed more than 8-fold HR efficiency than the control cell line; while knockdown of *BmXRCC4* gene (Fig. 4b) increased about 2-fold HR efficiency than the control cells.

### Epitope tagging of *BmTUDOR-SN* gene by CRISPR/Cas9-mediated knockin in silkworm cells

**Figure 5:**
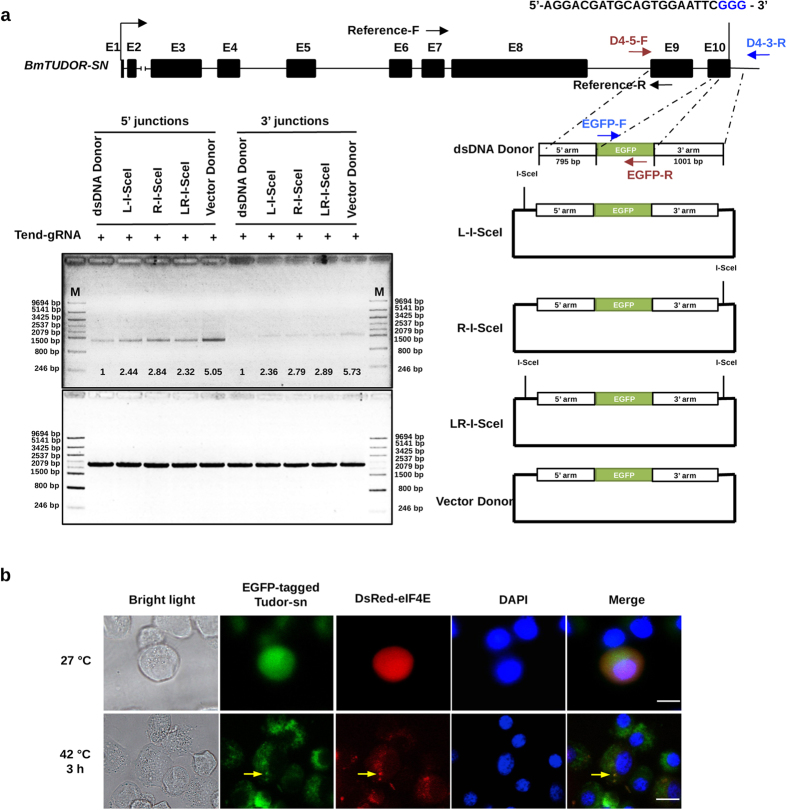
Epitope tagging of the *BmTUDOR-SN* gene. (**a**) Schematic overview depicting the exon 10 knockin strategy using different types of donors (dsDNA donor, cycle plasmid with an I-*Sce*I site on the flanking of left homologous arm, cycle plasmid with an I-*Sce*I site on the flanking of right homologous arm, cycle plasmid with two I-*Sce*I sites on the flanking of left and right homologous arms both, vector donor) and genomic DNA PCR analysis. Exons were indicated by black boxes, and target for gRNA of *BmTUDOR-SN* gene are indicated by a short straight line. The primers for 5′ junction (D4-5-F, EGFP-R), 3′ junction (EGFP-F, D4-3-R), and reference (Reference-F, Reference-R) amplification were marked by red, blue, and black arrows, respectively. Primer sequences were listed in Table S5. Genomic DNA PCR was performed at 7 days post transfection of the donors as indicated. The PCR products were diluted by a 2 fold serial dilution to be used for quantification of gel images with Image J software. Correct integration was calculated as fold shown in the upper gel by comparison with PCR products (dsDNA donor was set as 1 fold), with normalization to the corresponding reference PCR bands. The agarose gel images were representatives from repeated three independent experiments. The numbers below the PCR bands represent mean fold from the three repeats. (**b**) Images of knock-in cells under normal culture condition and stress condition. The knock-in BmN4 cells were enriched by a series of limiting dilutions after transfection with CRISPR/Cas9 system, plasmid donors. The enriched cells were transfected with DsRed-eIF4E expression vector^19^ and suffered from heat shock for 3h at 42 °C 3 days after transfection. The cells were visualized by a fluorescent microscope. *Scale bar*, 20 μl.

**Figure 6:**
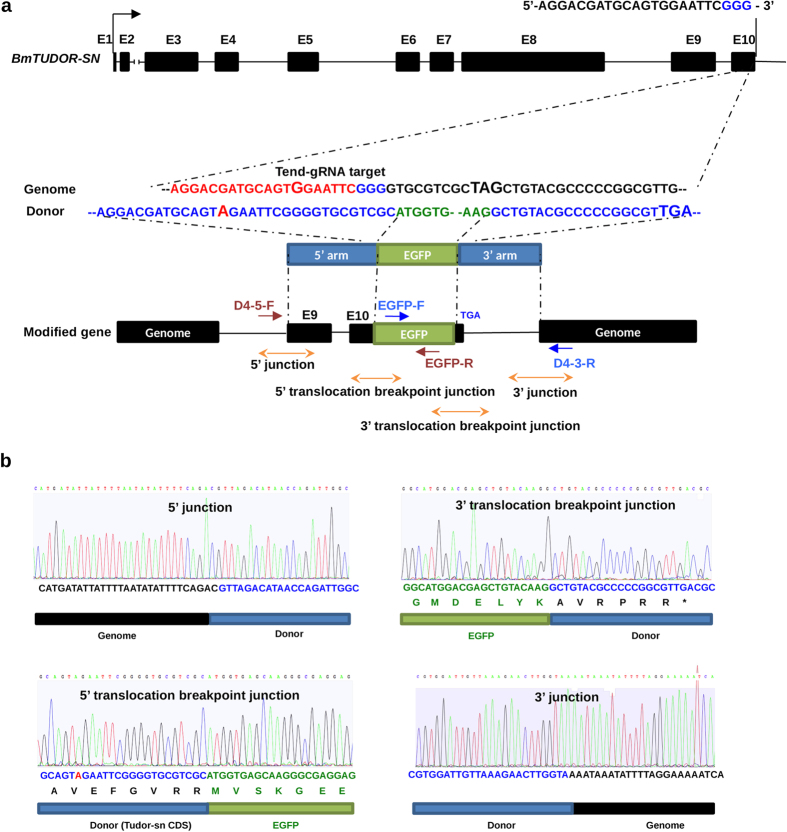
Sequencing of the modified *BmTUDOR-SN* gene. (**a**) A schematic representation of the *BmTUDOR-SN* gene and donor consisting of homologous arms and EGFP coding region. The gRNA targeting sequence was marked by red, and PAM was marked by blue. A guanine for modification was bold in the gRNA-targeting site in the genome sequence. The mutated nucleotide adenine was bold and red-marked in the donor sequence. The stop codons in genome or donor were bold. DNA fragments for sequencing were amplified by the primer sets, red-marked primers for 5′ junction and 5′ translocation breakpoint junction, blue-marked primers for 3′ junction and 3′ translocation breakpoint junction. (**b**) Chromatograms of DNA sequences of 5′ junction, 5′ translocation breakpoint junction, 3′ junction and 3′ translocation breakpoint junction in the modified *BmTUDOR-SN* gene. The homologous arms were marked by blue. EGFP coding sequence was marked by green. Genome was marked by black.

NHEJ-related gene knockout increased HR efficiency in the silkworm cells. Therefore, we expected that HR could be exploited for epitope tagging endogenous genes by gene conversion with designed donors. We designed a gRNA targeting exon 10 of the *BmTUDOR-SN* gene (Fig. 5a), which was found to encode a protein participating to stress granule formation^19^. Besides, we sought to target an *EGFP* gene to *BmTUDOR-SN* gene locus, using a donor containing an EGFP coding sequence flanked by a 795 bp 5′ and a 1001 bp 3′ homologous arms matching to the genome regions side of the DSB cut by Cas9 as shown in Fig. 5a. To avoid donor digestion by Cas9, we made a mutation (G to A) at the gRNA-targeting site in the donor (Fig. 6a). To detect the precise genome editing, we reduced the nucleotides, TA, at 3′ arm. Therefore, the stop codon was removed and another stop codon (TGA) downstream of EGFP-coding sequence in the donors would be recognized for translation stop. Previously, we found that I-*Sce*I-induced linearization of targeting vectors in silkworm cells increased the efficiency of gene targeting^10^. Here, we designed five kinds of donors (Fig. 5a), which were a dsDNA donor (the PCR-based HR donor), L-I-SceI, R-I-SceI, LR-I-SceI, and a plasmid donor. All donors were subjected to digestion by the purchased I-*Sce*I enzyme *in vitro* and donors containing I-*Sce*I sites were successfully cleaved into linearized plasmid DNAs (Fig. S3), verifying that I-*Sce*I sites were inserted into the corresponding donors. We co-transfected with Tend-gRNA expression vector (the corresponding gRNA sequence was listed in Table S2), Cas9 expression vector (Fig. S1), donors (Fig. 5), and the I-*Sce*I expression vector (Fig. 4a) into BmLig IV-KO cells. Genomic DNA PCR was performed after transient transfection to evaluate HR efficiency. As shown in Fig. 5a, using primer sets (Table S5) to amplify the 5′ junctions and 3′ junctions of the integrations, separately, we found all donors were available for CRISPR/Cas9-mediated gene targeting. Besides, the plasmid vector induced the highest efficiency of integration (up to more than 5 fold compared with the dsDNA donor). Moreover, all integration events were detectable but low efficient, because of small number of cells showing EGFP signals (Fig. S4a) and very weak bands of BmTudor-sn-fused EGFP protein from knock-in events detected in western blot (Fig. S4b). To exclude that the EGFP positive cells were formed by non-specific integration of EGFP-coding DNA in the genome, we did knock-in experiments without gRNA transfection. As shown in supplemental Figure S5, BmN4 cells transfected with only donors showed no EGFP signal. These data indicated that false-positive signals could be avoided by using our protocols including gRNA and donor design for knock-in experiments. Subsequently, we investigated the 5′ junction and 3′ junction by cloning the PCR products and sequenced (Fig. 6b), confirming that precise genome editing was achieved. Besides, to confirm the accessibility of CRISPR/Cas9-mediated knockin in silkworm cells, we designed donors targeting to other three loci in *BmTUDOR-SN* gene. As shown in supplemental Figure S5, positive bands representing 5′ and 3′ junctions with expected sizes were found in all the three knock-in experiments by genomic DNA PCR using the primers listed in Table S5. These data demonstrated that CRISPR/Cas9-mediated accurate gene targeting was available for silkworm cells.

To validate that the tagged endogenous Tudor-sn protein was involved in stress granule formation, we made limiting dilutions of BmN4 cells after co-transfection with CRISPR/Cas9 system and plasmid donor, followed by transfection with DsRed-fused eIF4E (a stress granule marker protein^19^) expression vector into the enriched cells. Then, the cells were suffered from heat shock treatment at 42 °C for 3 h and observed by a fluorescence microscope (Fig. 5b). After heat shock, the tagged Tudor-sn aggregated into stress granules marked by eIF4E protein, verifying that silkworm endogenous protein tagged by CRISPR/Cas9-mediated knockin was functional.

In addition, recent reports^20^,^21^,^22^ demonstrated that mutant Cas9 (D10A), which functioned as a nickase, could reduce off-target effects by using a paired gRNAs to improve CRSPR/Cas9 nuclease specificity. Therefore, making use of the paired gRNAs and Cas9 D10A might increase HR-mediated knockin efficiency. To estimate gene targeting mediated by Cas9 D10A, we chose site 3 of *BmTUDOR-SN* gene for the target and designed a paired gRNAs around site 3 as shown in supplementary Figure S7. The wild type of Cas9 was used for positive control and the Cas9 (dCas9) with double mutations (D10A and H840A) was used for negative control, because dCas9 could bind to target sites in defect of the cutting activity^23^. BmN4 cells were transfected with the modified or wild type CRISPR/Cas9 systems and Donor 4 followed by genomic PCR to compare the knockin efficiency. However, as shown in supplementary Figure S7c, the Cas9 D10A only slightly increased knockin efficiency, compared with the wild type.

### Simple and effective knockin by using short homologous arms in donors

**Figure 7:**
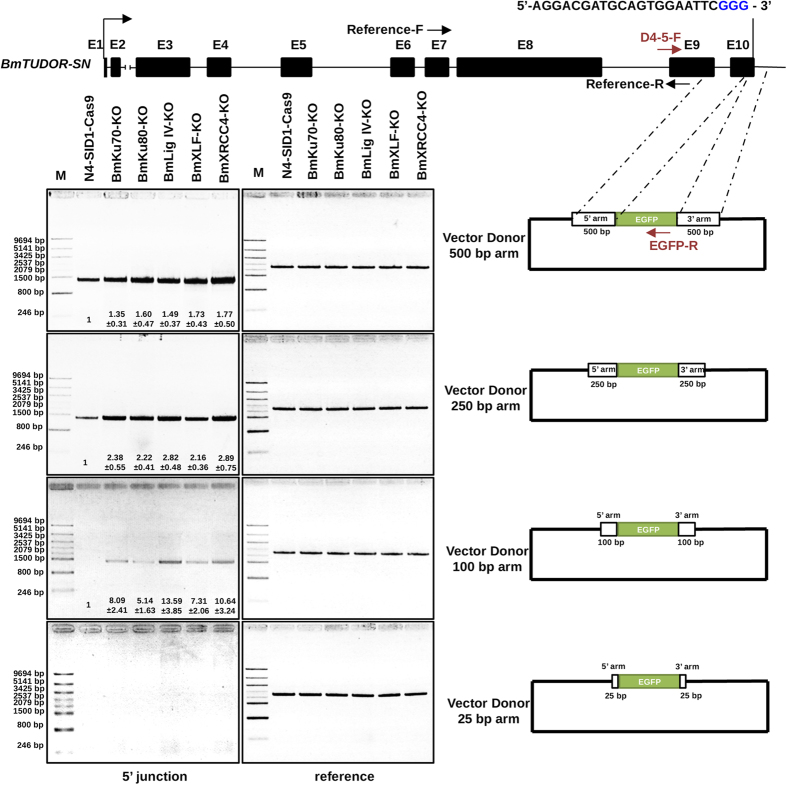
Higher efficiency of HR-mediated gene targeting in NHEJ-deficient cells using different length of homologous arms. Vector donors containing short homologous arms were used for *BmTUDOR-SN* gene editing of EGFP. Donors (300 ng/well each), pie2FW-Cas9 (300 ng/well), and Tend-gRNA expression vector (300 ng/well) were transfected into the indicated cells (1 × 10^5^ cells/well). Genomic DNA PCR was performed at 7 days post transfection of the donors as indicated. The gel images were analyzed by Image J to quantify the PCR products, with normalization to the reference bands. The PCR product from N4-SID1-Cas9 cells transfected with indicated donors was set as 1 fold. The agarose gel images were representatives from repeated three independent experiments. The numbers below the PCR bands represent mean fold ± S. D. from the three repeats.

We asked whether short homologous arms could be designed in donors for CRISPR/Cas9-mediated gene targeting in silkworm cells. We created plasmid donors with 500, 250, 100, or 25 bp homology, respectively, on either side of the DSB in genome cut by Cas9 for C-terminal tags of BmTudor-sn protein. We then performed the same knock-in experiments in NHEJ-deficient cells and also in only Cas9 expression cells with the donors containing designed short homology arms. The genomic DNA PCR (Fig. 7) indicated that CRISPR/Cas9-mediated gene targeting was achieved in NHEJ-deficiency cells and only Cas9 expression cells. Moreover, knock-in events were not found in the cells transfected with 25-bp homologous arms of donor, in spite of NHEJ-deficient cells. We quantified the intensity of the PCR bands by normalization with the intensity of the reference bands. NHEJ-deficient cells showed higher HR efficiency than the control cells with 500-bp, 250-bp, and 100-bp homologous arms of donors, such as up to 13. 59 fold in the BmLig IV-KO cells. These data suggested that in future, using the NHEJ-deficient cells, we could design as short as 100-bp homologous donors, which might be constructed by simple PCR amplification with primers consisting of partial sequence matching to homologous regions in interesting genes and partial sequence from tag protein or point mutated protein genes, for CRISPR/Cas9-mediated genome editing.

## Discussion

In this study, we validated the roles of NHEJ-related factors in the silkworm cells by a reporter cell line for detecting NHEJ activity. Subsequently, we constructed an All-In-One vector for expression of Cas9 and gRNAs targeting NHEJ-related factor genes to make NHEJ-deficient cells. After puromycin selection, we got the cell populations, which consisted of mutant cells and wild type cells. Interestingly, we found the knock-out cells had higher efficiency of knockin mediated by HR pathway, compared with normal cells. Unexpectedly, the knock-out cells had stronger activity of gene conversion than RNAi-mediated NHEJ-related gene knockdown cells (Fig. 4). It was possible that the remaining proteins in the knockdown cells functioned for NHEJ, and competed with HR factors for the DSBs. Therefore, using the All-In-One vector to knock out genes in silkworm cells may solve the situation, in which some genes were tough to be knocked down by corresponding dsRNAs. Additionally, it can be beneficial to purify mutant cells by taking the method of limiting dilution combined with western blot using specific anti-bodies. Furthermore, we successfully constructed BmTudor-sn mutated silkworm cells confirmed by western blot using Anti-BmTudor-sn antibody (Fig. S8). Thus far, there is no report about how to create gene knockout cell lines in silkworm, *Bombyx mori*. Our All-In-One vector is an optimal tool for making silkworm gene knockout cells to study gene functions in cellular signal pathways or cell physiology.

To improve knock-in frequency in a homology-dependent manner is our goal in this study. Knockdown and knockout of NHEJ-related factors could increase HR efficiency in the silkworm cells (Fig. 4). Moreover, NHEJ-deficient cells showed higher knock-in efficiency than BmN4 cells, especially by using short homologous arms in the donors (Fig. 7). In the HR assay by co-transfection with HR reporter into silkworm cells, the highest HR efficiency was found in BmXRCC4-KO cells (Fig. 4c). The BmXRCC4-KO cells also showed the highest HR efficiency in the knock-in experiments by using donors with 500 bp and 250 bp targeting arms. By using donors containing 100 bp targeting arms, the BmXRCC4-KO and BmLig IV-KO cells both showed high HR efficiency, which was even higher in BmLig IV-KO cells. In the NHEJ pathway, Lig IV and XRCC4 forms complex to complete DNA ligation and they are the key components in vertebrates^24^. In the silkworm cells, we also demonstrated that these two factors were critical for the function of NHEJ pathway. In the future, other NHEJ-related factor homologs, like DNA-PKs, Artemis, Pol X members, Polynucleotide kinase (PNK), etc. (see the review^24^), should be functionally analyzed in silkworm cells to further understand the mechanism of silkworm NHEJ pathway.

We performed CRISPR/Cas9-mediated knockin of *EGFP* gene to tag the endogenous gene, *BmTUDOR-SN.* The targeting efficiency was very low in normal BmN4 cells, while higher in NHEJ-deficient cells. However, when NHEJ pathway was suppressed, the frequency of knock-in cells was still not satisfactorily high in the silkworm cells, compared to other types of cells in different organisms^13^,^17^,^25^. The process of donor DNA integration into genome depends on several factors including the expression level of Cas9, the specific loci of targeting gene, length of homologous arms, and the probability of co-transfection of Cas9 expression vector, gRNA expression vector, and DNA donors into a cell simultaneously. Besides, the achievement of correct integration is also determined by specificity of gRNAs and donor structures. It therefore remains to be tested the efficiency of gene targeting in silkworm cells by using single stranded oligodeoxynucleotide (ssODN) as a donor, which was used in the knock-in experiments in silkworm embryo^5^,^8^. It may also be possible to improve the knock-in efficiency by double knockout or treble knockout of NHEJ-related genes or genes in other DNA repair pathway like single-strand annealing (SSA) and micro-homology-mediated end joining (MMEJ). To analyze knock-in cells in this report, we could isolate and enrich the knock-in cells by limiting dilutions or Fluoresence Activated Cell Sorting (FACS). Compared with the strategy of FACS, limiting dilution is very economic and available for the most of laboratories, which are using cultured cells. In addition, to facilitate enrichment of the genome-modified cells, we recommend knocking antibiotic gene expression cassettes in the target sites.

Our genome editing system presented in this study provides a simple and fast knock-out method by using All-In-One vector in silkworm cells. Besides, we found 100 bp homologous arms contained in donor vectors, which are accessible for donor construction, are feasible for knockin in NHEJ-deficient silkworm cells (Fig. 7). These results indicate that we are able to modify silkworm genome such as substitution, deletion, or single base modification, by using designed donors containing short homologous arms flanking the gRNA-targeting sites.

## Methods

### CRISPR/Cas9 system construction

Coding sequence (CDS) of mammalian codon-optimized *streptococcus pyogenes* Cas9 was amplified using KOD FX DNA polymerase (TOYOBO, Osaka, JP) from the JDS246 plasmid, which was a gift from Keith Joung (Addgene no. 43861), using the primers listed in supplementary Table S1. The Cas9 CDS was digested with *Not*I and cloned into an *EcroR*I/blunt-*Not*I site of pENTR^TM^11 vector (Invitrogen, Carlsbad, CA), which was modified by insertion of a nuclear localization signal at the 5′ upstream *EcoR*I site. The resulting plasmid was termed as pENTR-Cas9. The pENTR-Cas9 was used for Gateway® LR reaction with pPBO-FW-r^26^ to generate FLAG-fused Cas9 transgenic expression vector termed as pPBO-FW-r-Cas9. The pPBO-FW-r-Cas9 was used for construction of pPBO-FW-r-D10ACas9 and pPBO-FW-r-dCas9 by PCR amplification with the primers indicated in supplementary Table S1 and self-ligation with the linearized vectors. The pENTR-Cas9 was also used for LR reaction with pie2FW^26^ to generate FLAG-fused Cas9 vector, named as pie2FW-Cas9, for transient expression. The gRNA expression vector, psk-U6-gRNA, was constructed by replace of the fruit fly U6 promoter at pU6-BbsI-chiRNA (Addgene no. 45946) using silkworm U6 promoter^27^, which was amplified from silkworm BmN4 cell genomic DNA, using the primers in supplementary Table S1 (BmU6-2 promoter-F, BmU6-2 promoter-R). For all gRNAs, an additional 5′ G was added to improve U6 transcription. To construct the All-In-One vector as shown in Fig. 2b and supplementary Figure S2, pPBO-FW-r-Cas9 was linearized by PCR using the primers, followed by insertion with an U6-gRNA cassette amplified from psk-U6-gRNA using the phosphorylated primers (BmU6-2 promoter-F, chiRNA-R-polyT). To construct different gRNA expression vectors, either in All-In-One or in psk-U6-gRNA, the primer set (U6-promoter-R, corresponding gRNA forward primers in Table S2) should be used to linearize and self-ligate the gRNA expression vectors. After ligation, DpnI was used to digest methylated template DNA at 37 °C for 1 h. Successfully constructed gRNA expression vectors could be easily found by sequencing the colonies after DpnI treatment. All nucleotide sequences were confirmed by dye-terminator cycle sequencing (ABI PRISM).

### DNA donor preparation

A DNA fragment coding EGFP was cloned from pENTR-EGFP^28^ into an *EcoR*V site of pZERO2 (Invitrogen) using the primers (EGFP-full-F, EGFP-full-R-outstop). The homologous arms for *BmTUDOR-SN* gene targeting was amplified from genome DNA with the primers in supplementary Table S3 (T-end-uparm-F, T-end-uparm-R) for left arm, the primers in supplementary Table S3 (T-end-downarm-F, T-end-downarm-R) for right arm. The left and right arms were cloned into the linearized pZERO2-EGFP vectors amplified by the primers (EGFP-full-F, pZERO2-EcorV-R) and the primers (pZERO2-EcorV-F, EGFP-full-R) in supplementary Table S3, resulting two plasmids, pZERO2-leftarm-EGFP and pZERO2-EGFP-rightarm, respectively. Subsequently, the DNA fragments of 5′arm-EGFP and EGFP-3′arm were amplified from these two plasmids, respectively, followed by denaturation at 95 °C for 10 min and reannealing for producing leftarm-EGFP-rightarm DNA with cohesive ends. The leftarm-EGFP-rightarm DNA was amplified with the primers in supplementary Table S3 (T-end-uparm-F, T-end-downarm-R), and cloned into plits vector^29^ to generate the donor plasmid, termed as Tudor-sn-end-Donor. The 500 bp, 250 bp, 100 bp and 25 bp donors were constructed by amplifying appropriately sized fragments from Tudor-sn-end-Donor, using KOD FX DNA polymerase (TOYOBO) and cloning to pZERO2. The primers used for amplification these DNA fragments were listed indicatively in Table S3. To add I-*Sce*I recognizing sites in donor plasmids, the donors were amplified with the primers (plits-A036-ISceI-R2, plits-upT7-ISceI-F2) or the primers (plits-downT7-ISceI-R2, plits-A001-ISceI-F2) in supplementary Table S3 and self-ligated, resulting to generation of donors as shown in Fig. 5 with an I-*Sce*I site on the upstream of left arm or on the downstream of right arm, respectively.

### NHEJ and HR reporters

For NHEJ reporter, pENTR11-BmHP1α^30^ was recombined with the pIE2-mCherryW^26^ destination vector by using Gateway LR Clonase II Enzyme Mix (Invitrogen). The mCherry-HP1α sequence was amplified by PCR with the primers containing the I-*Sce*I recognition site “NHEJ I-SceI mCherry atg” and “NHEJ I-SceI IE2 polyA” (Table 1). The resulting PCR product possesses the recognition sites of I-*Sce*I at the both ends. The PCR product was subcloned into the *EcoR*V site of the pZERO2. The resulting plasmid was named pZERO-I-SceI-mCherry-HP1α. Next, the firefly luciferase gene was transferred into the pi2-GW^26^ destination vector by the Gateway LR reaction, generating pIE2-GFP-Luc. The I-*Sce*I recognition sequence was inserted between the *IE2* promoter and the GFP-luciferase fusion gene in pIE2-GFP-Luc by using the primers (NHEJ I-SceI linker a, NHEJ I-SceI linker b) in supplementary Table S1. The resulting plasmid was referred to as pIE2-I-SceI-GFP-Luc. Finally, the mCherry-HP1a sequence with the I-*Sce*I sites was amplified from pZErO-I-SceI-mCherry-HP1a by PCR using primers (NHEJ I-SceI mCherry atg, NHEJ I-SceI IE2 polyA) in supplementary Table S1. To make pIE2-I-SceI-mCherry-HP1α-I-SceI-GFP-Luc, the PCR product was digested by I-*Sce*I, and cloned into the I-*Sce*I cleaved pIE2-I-SceI-GFP-Luc. To generate a transgenic cell line expressing the NHEJ reporter in BmN4-SID1^31^ cells, the cassette of IE2-I-SceI-mCherry-HP1a-polyA-EGFP-Luciferase as shown in Fig. 1a was cloned into the *EcoR*V site of pPigGate vector^32^, which contained *piggyBac* transposon. After co-transfection of the modified pPigGate vector with pHA3PIG^18^ expressing *piggyBac* helper transposase under the control of the *B. mori* actinA3 promoter and puromycin selection, the BmN4-SID1 cells showed stable mCherry signals, and we named the cells as BmN4-SID1-NHEJ. For detecting HR efficiency, we used the same plasmids, which were constructed before for establishment of HR reporter cell lines^18^. The I-SceI expression vector was constructed before^10^.

### Cell culture and transfection

To establish knock-out cell lines, BmN4-SID1 cells were prepared in 24-wells at the density of 1 × 10^5^ cells/well with 500 μl IPL-41 medium (GIBCO-BRL, Grand island, NY) containing 10% fetal bovine serum at 27 °C overnight. After 16 h of incubation, 300 ng each of All-in-one vectors for knockout genes were co-transfected with 300 ng helper vector separately using the same procedures as described before^19^. To induce DNA breaks with CRISPR/Cas9 system for knock-in experiments (Figs 5 and 7), 300 ng pie2FW-Cas9 was co-transfected with 300 ng corresponding gRNA expressing vectors and 300 ng donors into the silkworm cells. For heat shock treatment and cell observation, the pie2FW-Cas9, Tend-gRNA expression vector, vector donor targeting to site 3 of *BmTUDOR-SN* gene, were co-transfected into BmN4 cells, followed by limiting dilution to enrich the knocked in cells. Then, the enriched cells were transfected with DsRed-eIF4E expression vector^19^. The cells were plated on the micro cover glasses and mounted with VectaShield (Vector Laboratories, Inc., Burlingame, CA, USA) at 3 days post transfection, and incubated at 42 °C for 3 h. Fluorescence microscopy was performed on a Biozere BZ-8000 fluorescence microscopy. For knockdown of endogenous genes (Figs 1d and 4b), the BmN4-SID1 cells with or without reporters were cultured in 24-wells at the density of 0.6 × 10^5^ cells with 100 ng/well different dsRNAs for 3 days, followed by transfection with 300 ng I-*Sce*I vector^18^ mixed with or without reporters, and subjected to Luc assay another 3 days later. Luc assay, dsRNA synthesis, RT-PCR and western blot were performed as previously described^19^. The antibody for BmTudor-sn was purified from the rabbit serum after injection with the peptide aa 874-aa 888 (Thermo Scientific).

### Cell proliferation assay

Silkworm cells indicated in Fig. 2f were cultured in a 96-well plate at 27 °C with a concentration of 0.5 × 10^4^ cells/well. At 3, 5, 7 and 10 days after incubation, 10 μl of solution in cell counting kit-8 (CCK8, DOJINDO Molecular TECHOLOGIES) was added to each well. The cells covered by foil paper then was incubated at 27 °C for 6 h. Details about the experimental procedures were described in the manual of CCK8. The absorbance at 450 nm was measured by a microplate reader (Bio-Red). Data are representative of three independent experiments performed in triplicate.

### Genomic DNA PCR

The cultured silkworm cells were digested using lysis buffer (50 mmol/L Tris-HCl PH 8.0, 100 mmol/L EDTA, 100 mmol/L NaCl, and 1% SDS) and incubated with working concentration of 0.25 mg/ml protein kinase K at 55 °C for 3 h. The genome DNA from the cell lysates were purified by phenol:chloroform, precipitated with ethanol, and resuspended in ddH_2_O. With 200 ng DNA in 50 μl reactions, genomic DNA PCR was performed using the primers listed in Table S5. KOD FX DNA polymerase was used in the standard 50 μl reaction system as described in the manufacturer’s manual. The PCR was performed in a thermocycler with the following protocol: 94 °C, 2 min; (98 °C, 10 sec; 60 °C, 30 sections; 68 °C, 2 min;) 35 cycles; hold at 4 °C. PCR products were analyzed on 1% agarose gels, and cloned into an *EcoR*V-cut pZERO2 for sequencing.

### T7EI cleavage assay

PCR amplicons from silkworm cell genomic DNA were purified with PCI/CIA treatment and precipitated with ethanol. Then, the PCR products were denatured and annealed in NEBuffer 2 (NEB) using a thermocycler with the following protocol: 95 °C, 5 min; 95–85 °C at −2 C/s; 85–25 °C at −0.1 °C/s; hold at 4 °C, subsequently digested with T7 endonuclease I (NEB, M0302S) for 30 min at 37 °C. The enzyme-DNA mixtures were subjected to 2.0% agarose gel electrophoresis, with the PCR products without T7EI treatment as the negative control. Quantification was based one relative band intensities obtained from Image J analysis. The indels were calculated based on the fraction of cleaved DNA (details were described in the previous report^33^).

### gRNA design and avoidance of off-target activities

For design of gRNAs, the target sites were selected with 5′-N_(21)_-GG, or 5′-G-N_(21)_-GG, or 5′-G-N_(20)_-GG in the exon regions of the indicated genes, following cloning and sequence of the target region. According to the recent report^34^, the CRISPR/Cas9 system tolerates three to five mismatches in the PAM-distal region. Therefore, to check gRNA target sites and potential off-target sites in silkworm genome, the sequences of 5′-N_(16)_-GG, or 5′-N_(17)_-GG were searched using BLAST algorithm on the SilkDB (http://silkworm.swu.edu.cn/silkdb/). Without any potential off-target sites in genome, we designed the primers (Table S2) for construction of gRNA expression vectors.

## Additional Information

**How to cite this article**: Zhu, L. *et al.* CRISPR/Cas9-mediated knockout of factors in non-homologous end joining pathway enhances gene targeting in silkworm cells. *Sci. Rep.* **5**, 18103; doi: 10.1038/srep18103 (2015).

## Supplementary Material

Supplementary Information

## References

[b1] Xia Q. *et al.* A draft sequence for the genome of the domesticated silkworm (*Bombyx mori*). Science 306, 1937–1940, doi: 10.1126/science.1102210 (2004). 15591204

[b2] Xia Q., Li S. & Feng Q. Advances in silkworm studies accelerated by the genome sequencing of *Bombyx mori*. Annu. Rev. Entomol. 59, 513–536, doi: 10.1146/annurev-ento-011613-161940 (2014). 24160415

[b3] Takasu Y. *et al.* Targeted mutagenesis in the silkworm *Bombyx mori* using zinc finger nuclease mRNA injection. Insect Biochem. Mol. Biol. 40, 759–765, doi: 10.1016/j.ibmb.2010.07.012 (2010). 20692340

[b4] Ma S. *et al.* Highly efficient and specific genome editing in silkworm using custom TALENs. PLoS One 7, e45035, doi: 10.1371/journal.pone.0045035 (2012). 23028749 PMC3445556

[b5] Ma S. *et al.* Multiplex genomic structure variation mediated by TALEN and ssODN. BMC genomics 15, 41, doi: 10.1186/1471-2164-15-41 (2014). 24438544 PMC3933007

[b6] Wang Y. *et al.* Site-specific, TALENs-mediated transformation of *Bombyx mori*. Insect Biochem. Mol. Biol. 55C, 26–30, doi: 10.1016/j.ibmb.2014.10.003 (2014). 25460511 PMC4408225

[b7] Wang Y. *et al.* The CRISPR/Cas system mediates efficient genome engineering in *Bombyx mori*. Cell Res. 23, 1414–1416, doi: 10.1038/cr.2013.146 (2013). 24165890 PMC3847576

[b8] Ma S. *et al.* CRISPR/Cas9 mediated multiplex genome editing and heritable mutagenesis of BmKu70 in *Bombyx mori*. Sci. Rep. 4, 4489, doi: 10.1038/srep04489 (2014). 24671069 PMC3967148

[b9] Liu Y. *et al.* Highly efficient multiplex targeted mutagenesis and genomic structure variation in *Bombyx mori* cells using CRISPR/Cas9. Insect Biochem. Mol. Biol. 49, 35–42, doi: 10.1016/j.ibmb.2014.03.010 (2014). 24698835

[b10] Mon H., Kusakabe T., Lee J. M., Kawaguchi Y. & Koga K. *In vivo* DNA double-strand breaks enhance gene targeting in cultured silkworm cells. Comp. Biochem. Physiol. B Biochem. Mol. Biol. 139, 99–106, doi: 10.1016/j.cbpc.2004.06.013 (2004). 15364292

[b11] Hsu P. D., Lander E. S. & Zhang F. Development and applications of CRISPR-Cas9 for genome engineering. Cell 157, 1262–1278, doi: 10.1016/j.cell.2014.05.010 (2014). 24906146 PMC4343198

[b12] Mali P. *et al.* RNA-guided human genome engineering via Cas9. Science 339, 823–826, doi: 10.1126/science.1232033 (2013). 23287722 PMC3712628

[b13] Maruyama T. *et al.* Increasing the efficiency of precise genome editing with CRISPR-Cas9 by inhibition of nonhomologous end joining. Nature Biotechnol. 33, 538–542, doi: 10.1038/nbt.3190 (2015). 25798939 PMC4618510

[b14] Chu V. T. *et al.* Increasing the efficiency of homology-directed repair for CRISPR-Cas9-induced precise gene editing in mammalian cells. Nature Biotechnol. 33, 543–548, doi: 10.1038/nbt.3198 (2015). 25803306

[b15] Beumer K. J., Trautman J. K., Mukherjee K. & Carroll D. Donor DNA Utilization during Gene Targeting with Zinc-finger Nucleases. G3, doi: 10.1534/g3.112.005439 (2013). PMC3618352 23550125

[b16] Beumer K. J. *et al.* Efficient gene targeting in *Drosophila* by direct embryo injection with zinc-finger nucleases. Proc. Natl. Acad. Sci. USA 105, 19821–19826, doi: 10.1073/pnas.0810475105 (2008). 19064913 PMC2604940

[b17] Bottcher R. *et al.* Efficient chromosomal gene modification with CRISPR/cas9 and PCR-based homologous recombination donors in cultured *Drosophila* cells. Nucleic Acids Res. 42, e89, doi: 10.1093/nar/gku289 (2014). 24748663 PMC4066747

[b18] Mon H., Lee J., Kawaguchi Y. & Kusakabe T. Double-strand breaks repair by gene conversion in silkworm holocentric chromosomes. Mol. Genet. Genomics 286, 215–224, doi: 10.1007/s00438-011-0640-1 (2011). 21842267

[b19] Zhu L. *et al.* Characterization of Tudor-sn-containing granules in the silkworm, Bombyx mori. Insect Biochem. Mol. Biol. 43, 664–674, doi: 10.1016/j.ibmb.2013.04.004 (2013). 23643815

[b20] Fu Y., Sander J. D., Reyon D., Cascio V. M. & Joung J. K. Improving CRISPR-Cas nuclease specificity using truncated guide RNAs. Nature Biotechnol. 32, 279–284, doi: 10.1038/nbt.2808 (2014). 24463574 PMC3988262

[b21] Shen B. *et al.* Efficient genome modification by CRISPR-Cas9 nickase with minimal off-target effects. Nature methods 11, 399–402, doi: 10.1038/nmeth.2857 (2014). 24584192

[b22] Ran F. A. *et al.* Double nicking by RNA-guided CRISPR Cas9 for enhanced genome editing specificity. Cell 154, 1380–1389, doi: 10.1016/j.cell.2013.08.021 (2013). 23992846 PMC3856256

[b23] Jinek M. *et al.* A programmable dual-RNA-guided DNA endonuclease in adaptive bacterial immunity. Science 337, 816–821, doi: 10.1126/science.1225829 (2012). 22745249 PMC6286148

[b24] Michael R. Lieber. The mechanism of double-strand DNA break repair by the nonhomolous DNA end-joining pathway. Annu. Rev. Biochem. 79, 181–211, doi: 10.1146/annurev.biochem.052308.093131 (2010). 20192759 PMC3079308

[b25] Ho T. T. *et al.* Targeting non-coding RNAs with the CRISPR/Cas9 system in human cell lines. Nucleic Acids Res. 43, e17, doi: 10.1093/nar/gku1198 (2015). 25414344 PMC4330338

[b26] Mitsunobu H. *et al.* construction of gateway-based destination vectors for detcting subcellular localization of proteins in the silkworm, *Bomyx mori*. J. Insect Biotechnol. Sericol. 75, 141–145 (2006).

[b27] Tanaka H. *et al.* shRNA expression plasmids generated by a novel method efficiently induce gene-specific knockdown in a silkworm cell line. Mol. Biotechnol. 41, 173–179, doi: 10.1007/s12033-008-9108-x (2009). 18821064

[b28] Zhu L. *et al.* A MC motif in silkworm Argonaute 1 is indispensible for translation repression. Insect Mol. Biol. 22, 320–330, doi: 10.1111/Imb.12023 (2013). 23521747

[b29] Zhu L. *et al.* Molecular cloning of *BmTUDOR-SN* and analysis of its role in the RNAi pathway in the silkworm, *Bombyx mori* (Lepidoptera: Bombycidae). Appl. Entomol. Zool. 47, 207–215, doi: 10.1007/S13355-012-0109-7 (2012).

[b30] Tatsuke T. *et al.* Roles of Piwi proteins in transcriptional regulation mediated by HP1s in cultured silkworm cells. PLoS One 9, e92313, doi: 10.1371/journal.pone.0092313 (2014). 24637637 PMC3956929

[b31] Mon H. *et al.* Soaking RNAi in *Bombyx mori* BmN4-SID1 cells arrests cell cycle progression. J. Insect Sci. 13, 155, doi: 10.1673/031.013.15501 (2013). 24773378 PMC4015410

[b32] Tatsuke T. *et al.* Tightly controlled tetracycline-inducible transcription system for explosive gene expression in cultured silkworm cells. Arch. Insect Biochem. Physiol. 82, 173–182, doi: 10.1002/arch.21083 (2013). 23371880

[b33] Cong L. *et al.* Multiplex genome engineering using CRISPR/Cas systems. Science 339, 819–823, doi: 10.1126/science.1231143 (2013). 23287718 PMC3795411

[b34] Kuscu C., Arslan S., Singh R., Thorpe J. & Adli M. Genome-wide analysis reveals characteristics of off-target sites bound by the Cas9 endonuclease. Nature Biotechnol. 32, 677–683, doi: 10.1038/nbt.2916 (2014). 24837660

