# Supplementary material for: CRISPR/Cas9-mediated knockout of factors in non-homologous end joining pathway enhances gene targeting in silkworm cells
Source: Sci Rep. 2015 Dec 10;5:18103. doi: 10.1038/srep18103 (PMC4674802; doi:10.1038/srep18103)
Supplement: Supplementary Information [file srep18103-s1.pdf]

Supplementary information

CRISPR/Cas9-mediated knockout of factors in non-homologous end joining pathway enhances gene targeting in silkworm cells

Li Zhu, Hiroaki Mon, Jian Xu, Jae Man Lee, Takahiro Kusakabe*

Laboratory of Insect Genome Science, Kyushu University Graduate School of Bioresource and Bioenvironmental Sciences, Hakozaki 6-10-1, Fukuoka 812-8581, Japan

* Correspondence author: T. Kusakabe
Tel./fax: +81-92-642-2842.
E-mail: kusakabe@agr.kyushu-u.ac.jp

Contents:

Supplementary Figure S1-8
Supplementary Table S1-5

Supplementary Figure S1

a

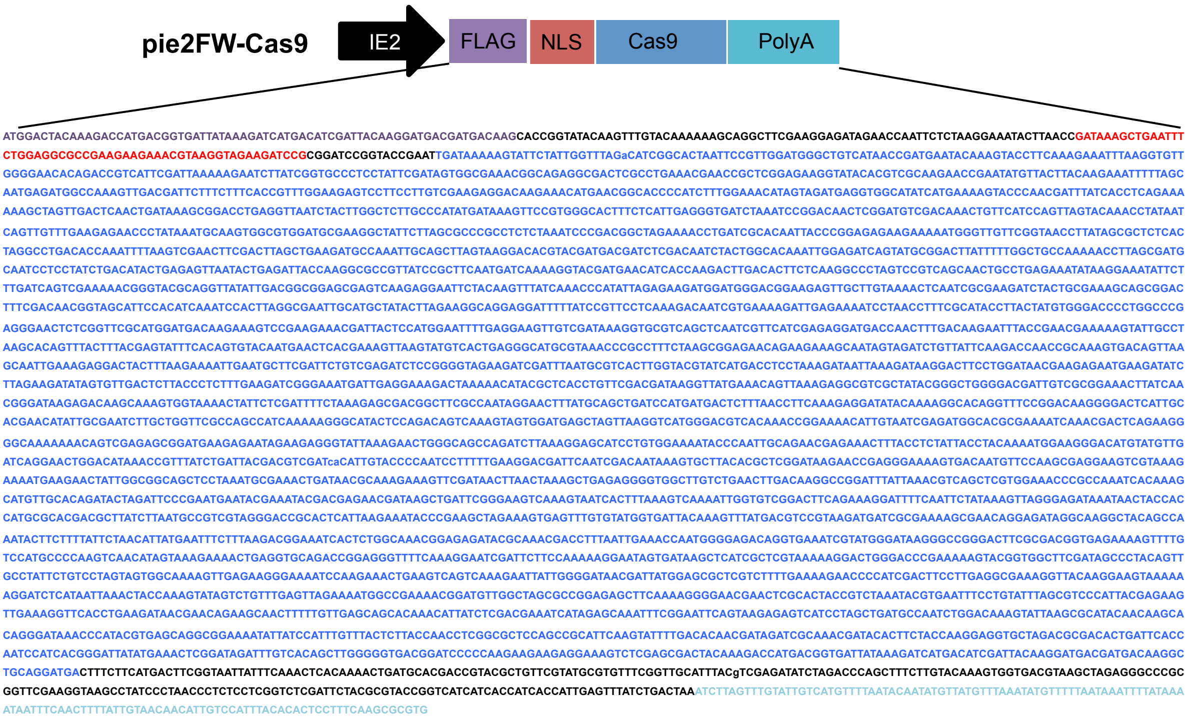

b

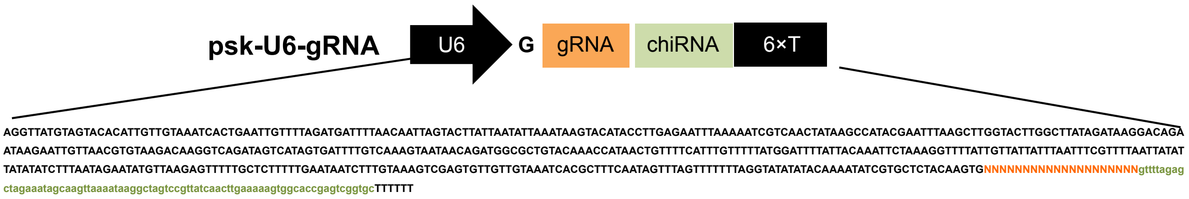

Supplementary Figure S1. Sequence of Cas9 expression and gRNA expression vectors. (a) Cas9 was expressed under a IE2 promoter; (b) gRNA was expressed under silkworm U6 promoter.

Supplementary Figure S2

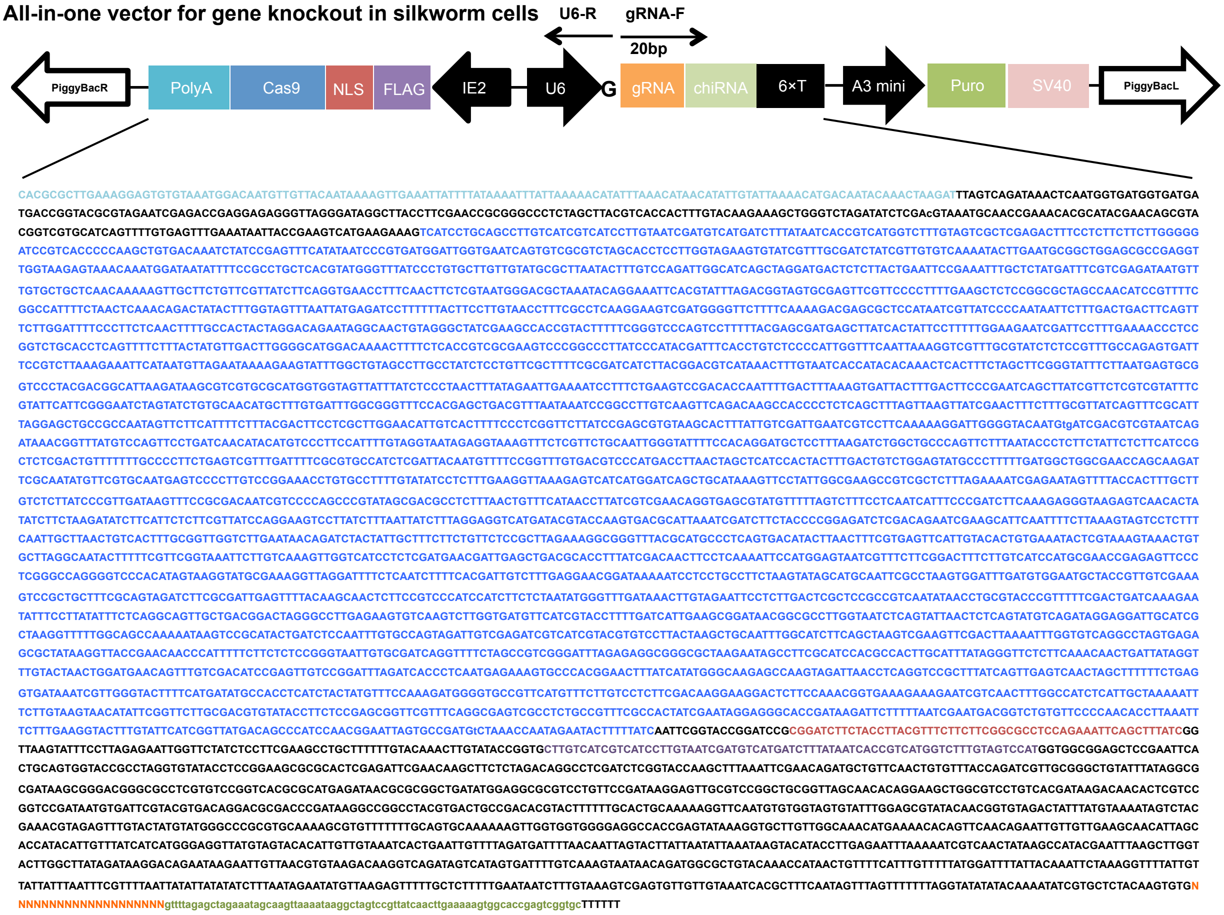

Supplementary Figure S2. Sequence of the All-In-One vector. A cassette for Cas9 expression and a cassette for gRNA expression were inserted into the middle of *PiggyBac* arms. A cassette for puromycin expression was used for cell selection.

Supplementary Figure S3

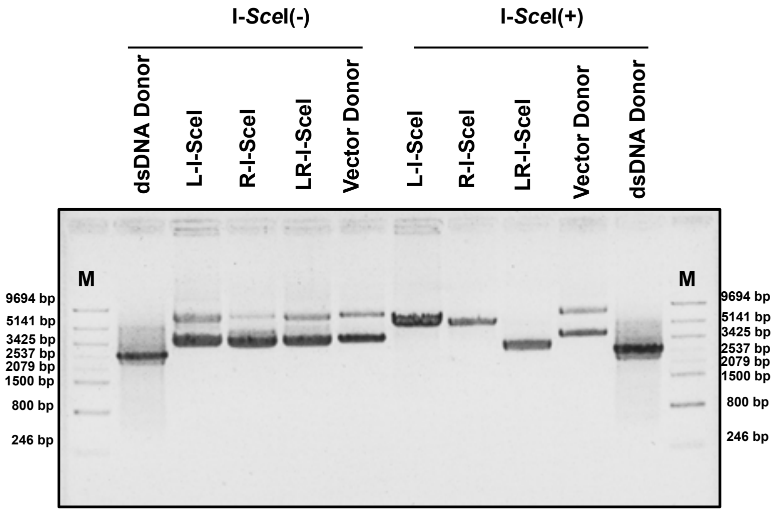

Supplementary Figure S3. Gel image of plasmid DNAs and DNA fragments used for transfection in knockin experiments. To confirm I-*Sce*I sites were inserted in plasmid donors or nor, constructed plasmids (L-I-SceI, R-I-SceI, LR-I-SceI, and Vector donor) were digested *in vitro* by I-*Sce*I for 15 min at 37 °C, followed by gel electrophoresis. PCR products (dsDNA Donor) was used as a control for evaluation of DNA size.

Supplementary Figure S4

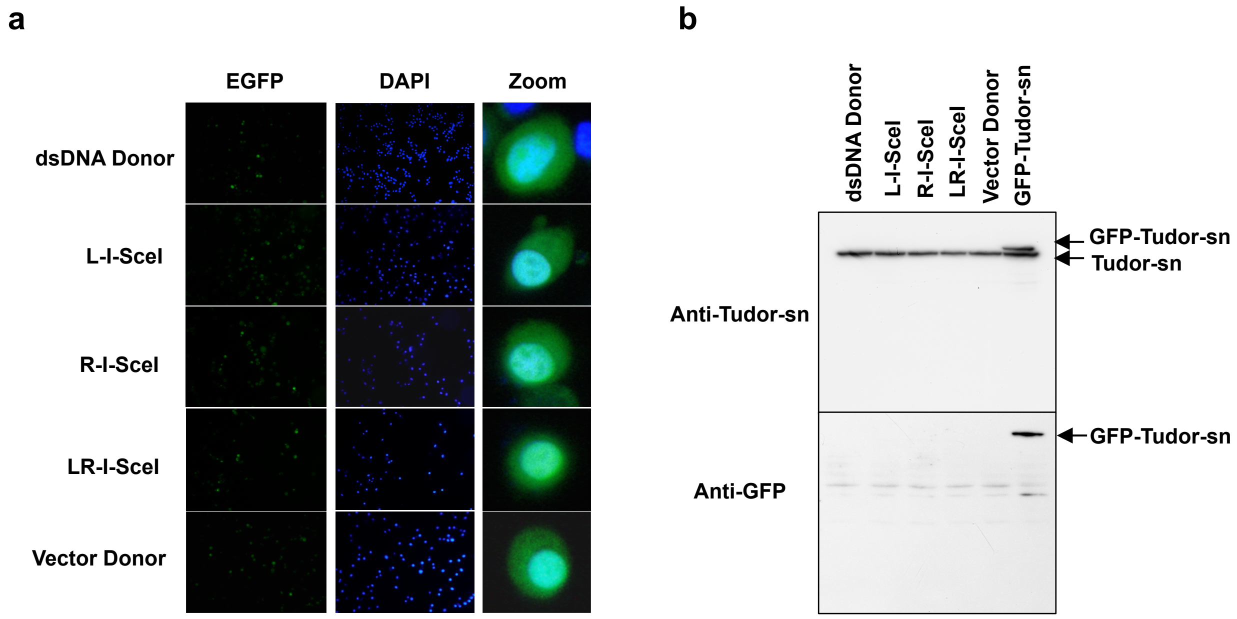

Supplementary Figure S4. Expression of EGFP-fused BmTudor-sn. (a) Knock-in positive cells were visualized by a fluorescent microscope. (b) Western blot was performed to detect the fusion protein.

Supplementary Figure S5

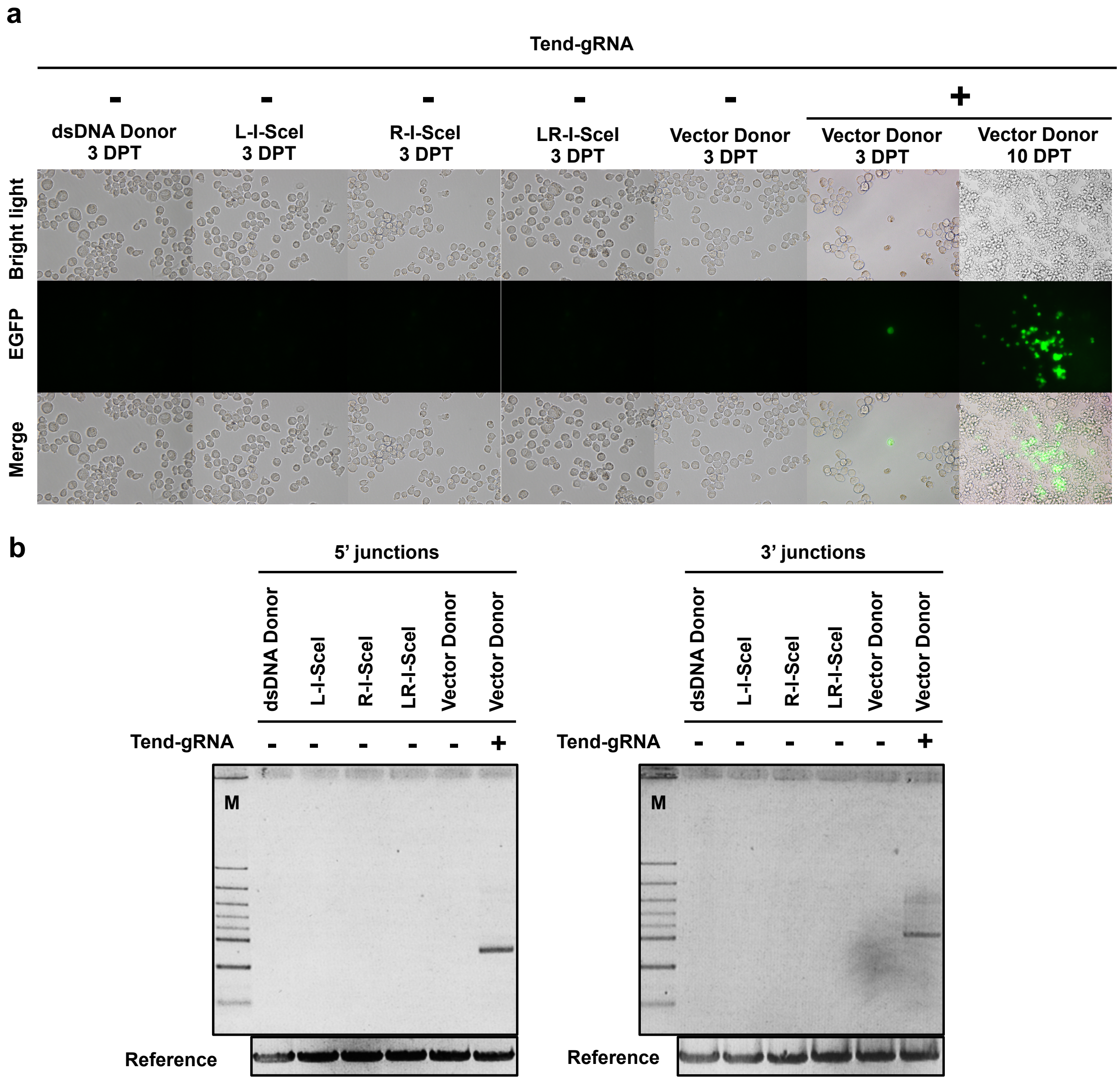

Supplementary Figure S5. Random integration was not found in the cells transfected with only donors. (a) Images of BmN4 cells transfected with pie2FW-Cas9, donors and Tend-gRNA expression vector (+), or without Tend-gRNA expression vector (-). There was no signal in the cell without Tend-gRNA transfection 3 days post transfection (DPT). (b) Genomic DNA PCR was performed from the cells transfected with pie2FW-Cas9, donors and Tend-gRNA expression vector (+), or without Tend-gRNA expression vector (-), after fluorescence microscope observation. 5’ junction and 3’ junction were not amplified in the genomes of the cells without transfection of gRNA expression vector.

Supplementary Figure S6

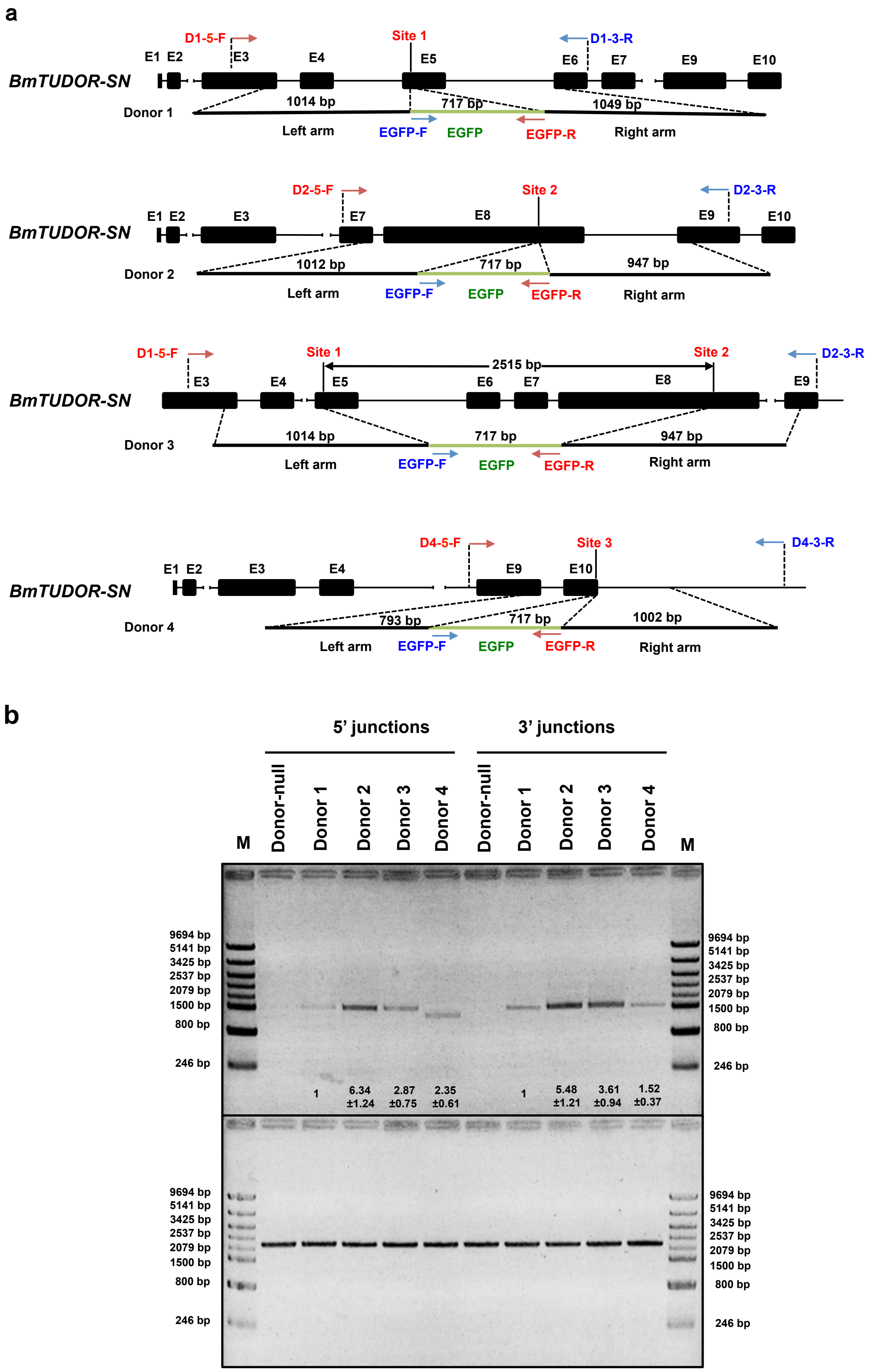

Supplementary Figure S6. Knock-in of EGFP gene at three different sites of *BmTUDOR-SN* gene. (a) Schematic illustration of knock-in strategies for targeting to the sites in *BmTUDOR-SN* gene with four kinds of DNA donors (plasmids). Exons were indicated by black boxes, and target for gRNA of *BmTUDOR-SN* gene are indicated by a short straight line. The primers for 5' junction and reference amplification were marked by red and black arrows, respectively. Primer sequences were listed in Table S5. (b) Genomic DNA PCR was performed at 7 days post transfection of the donors as indicated. The gel images were analyzed by Image J to quantify the PCR products, with normalization to the reference bands. The PCR product from BmN4-SID1-Cas9 cells transfected with donor 1 was set as 1 fold. The agarose gel images were representatives from repeated three independent experiments. The numbers below the PCR bands represent mean fold ± S. D. from the three repeats.

Supplementary Figure S7

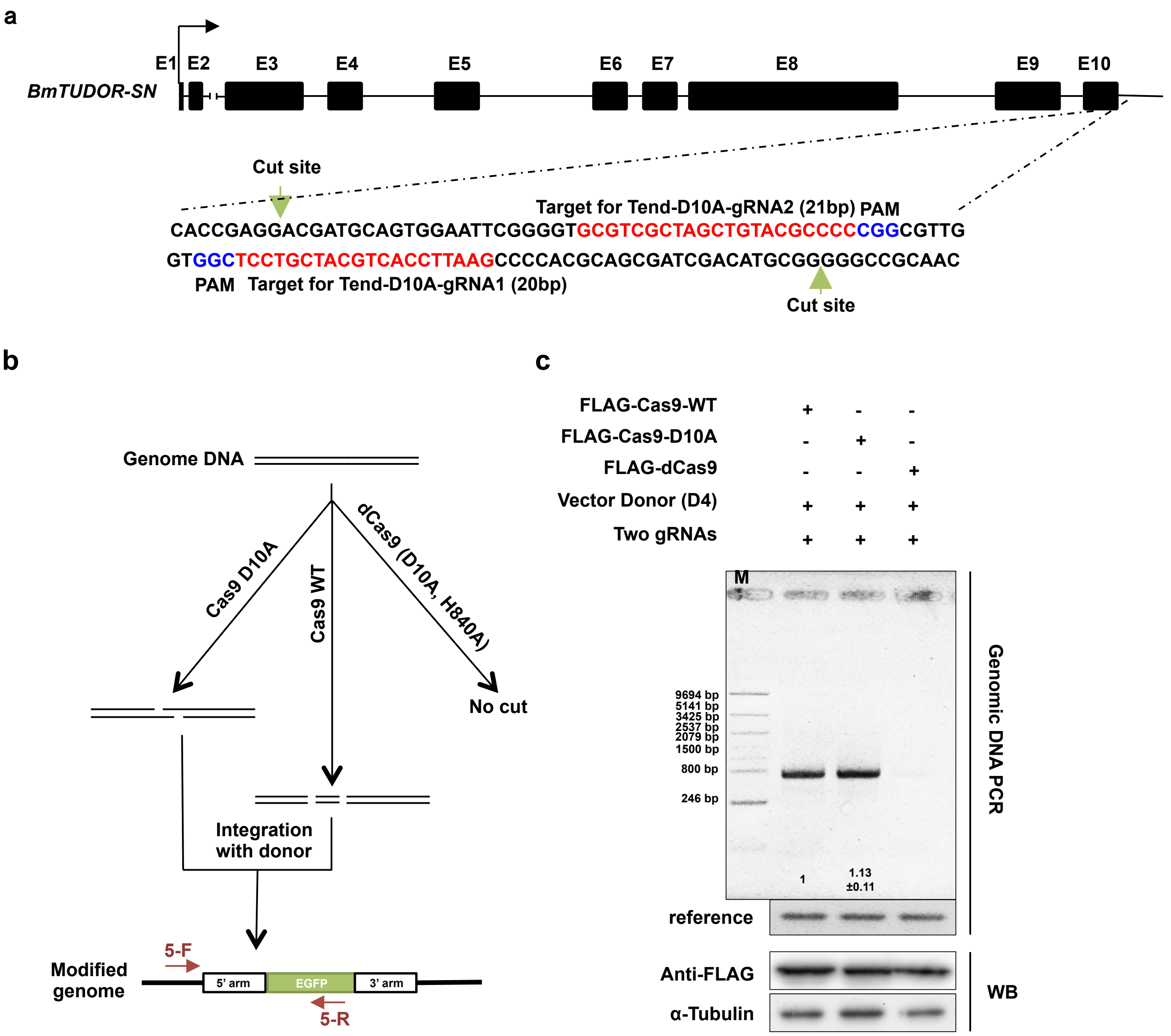

Supplementary Figure S7. Comparison of knock-in efficiency between wild type of Cas9 and mutant Cas9 proteins (a) Schematic illustration of knock-in strategies for targeting to the site 3 in *BmTUDOR-SN* gene with paired gRNAs and Donor 4 plasmid. The gRNA targeting sequence was marked by red, and PAM was marked by blue. The cut sites by Cas9 D10A were marked by green arrow heads. (b) Graphic representations of *BmTUDOR-SN* gene editing using different types Cas9 proteins. The primers for 5' junction amplification were marked by red. Primer sequences were listed in Table S5. (c) Genomic DNA PCR was performed at 7 days post transfection. The gel images were analyzed by Image J to quantify the PCR products, with normalization to the reference bands. The PCR product from BmN4 cells transfected with wild type Cas9 expression vector was set as 1 fold. The agarose gel images were representatives from repeated three independent experiments. The numbers below the PCR bands represent mean fold from the three repeats. Western blot was performed to detect Cas9 protein levels. α-Tubulin was used for loading control.

Supplementary Figure S8

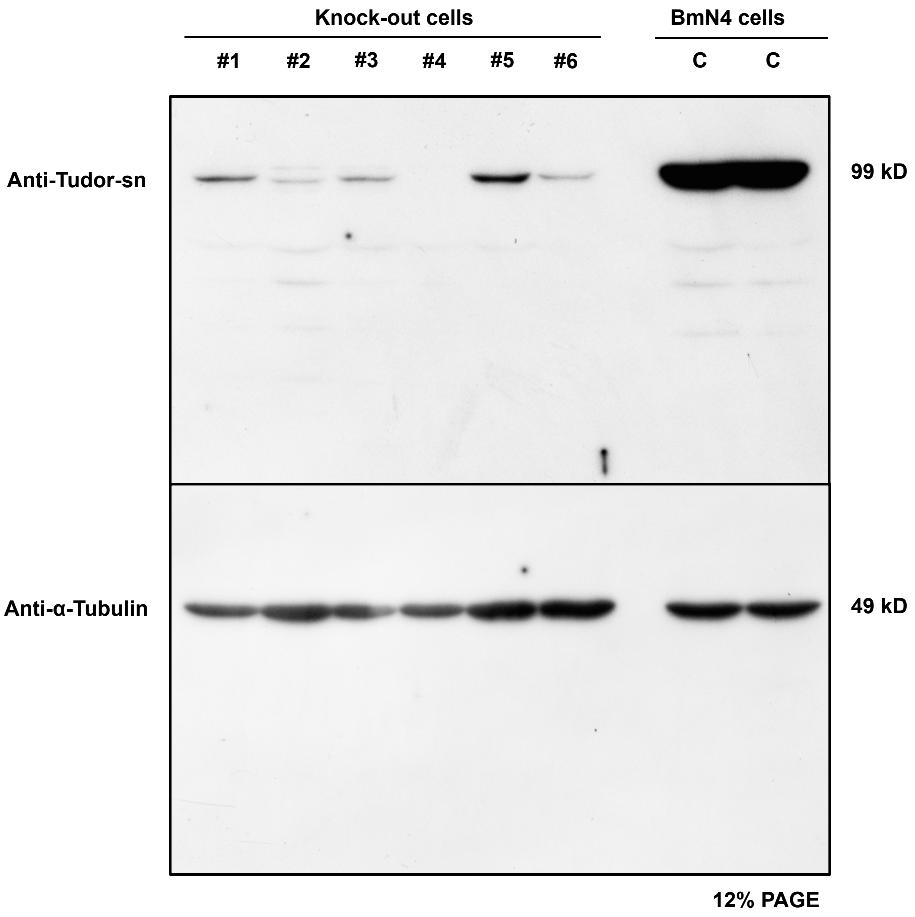

Supplementary Figure S8. Western blot was performed to detect BmTudor-sn protien in knock-out cells and normal BmN4 cells. The BmN4 cells were transfected with All-In-One vector targeting to the site 2 of *BmTUDOR-SN* gene, followed by limiting dilutions for isolation of mutant cells. After several times of limiting dilution, six cell lines were subjected to western blot. C is normal BmN4 cells served as the negative control.

Supp. Fig. 1d. Supplementary full length DNA gel.

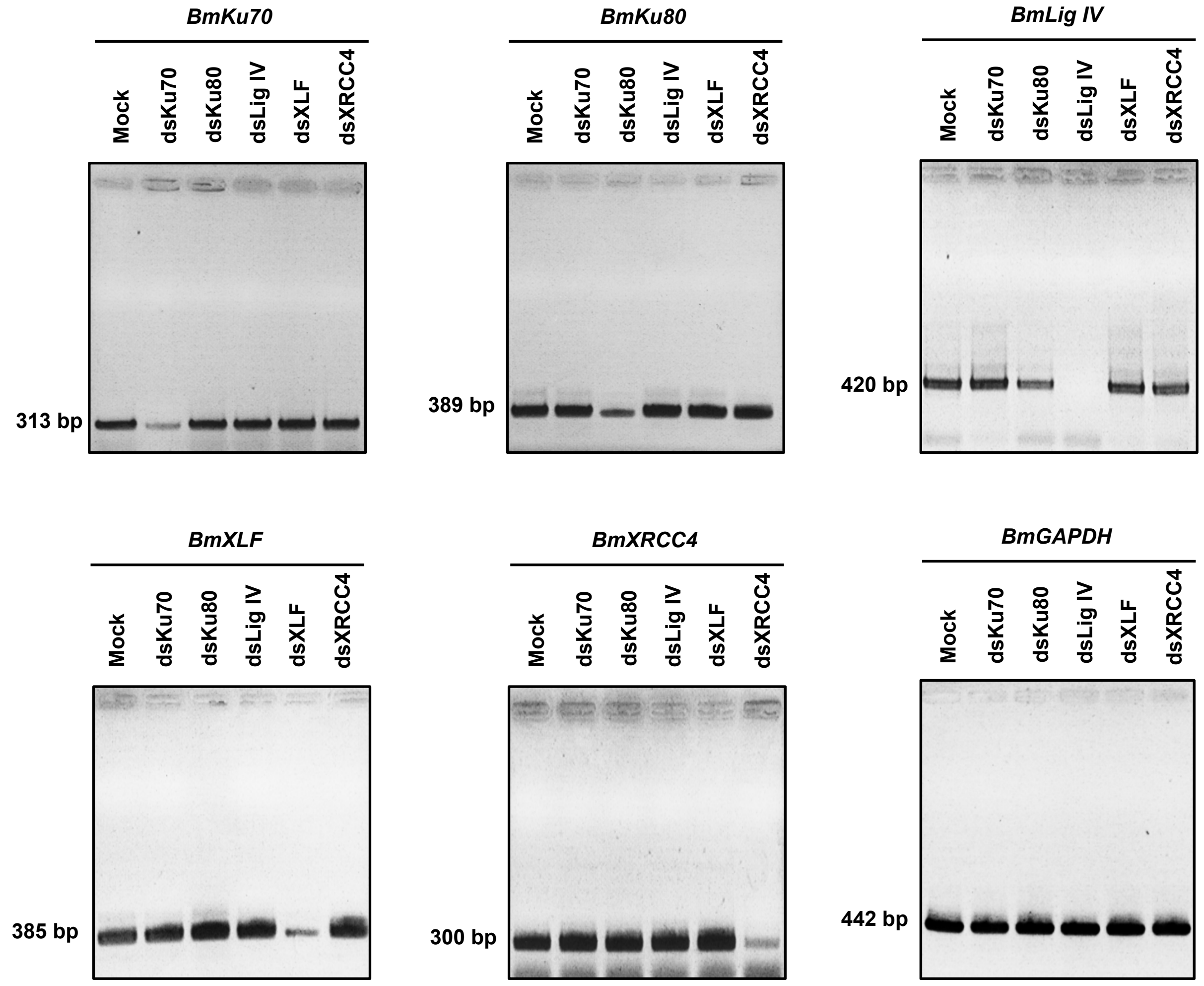

Supp. Fig. 2d. Supplementary full length western blot.

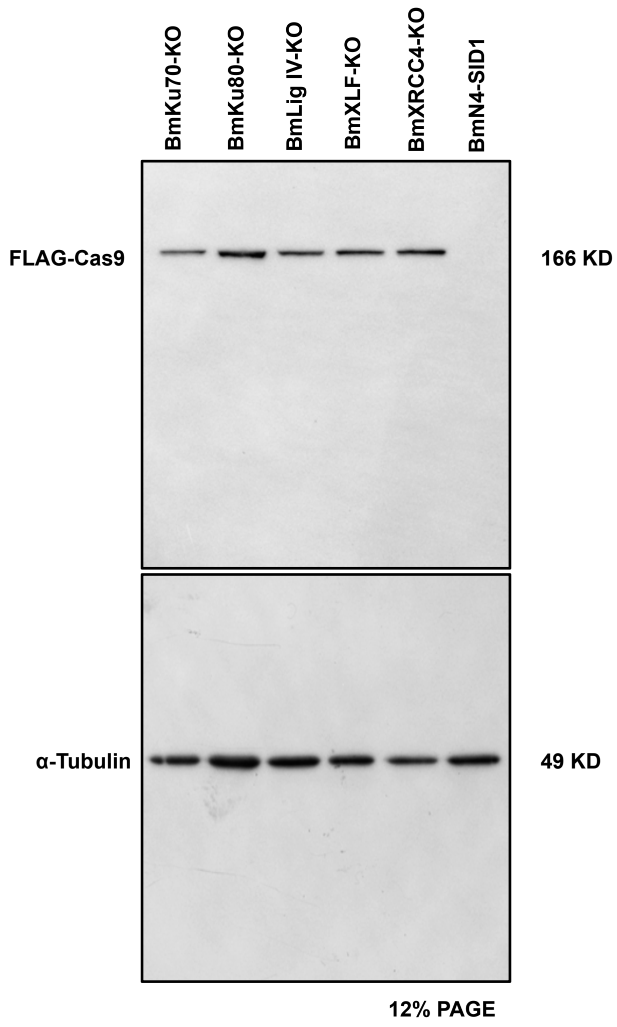

Supp. Fig. 2e. Supplementary full length DNA gel.

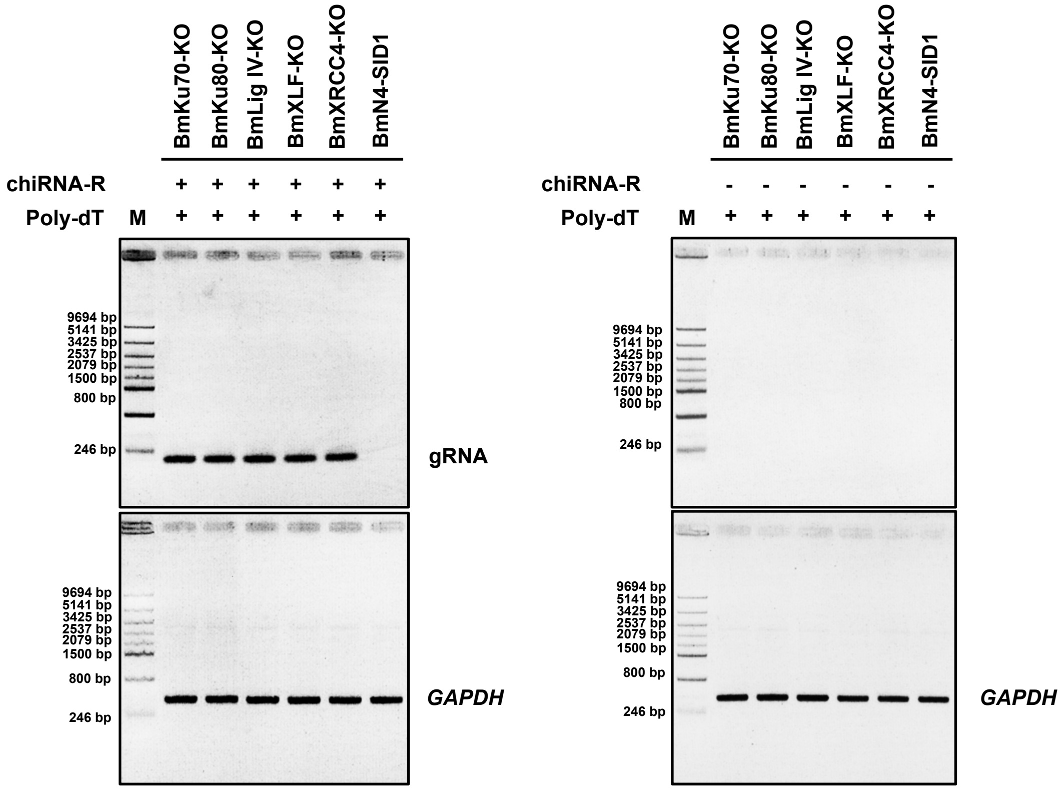

Supp. Fig. 4b and Fig. 4c. Supplementary full length western blot.

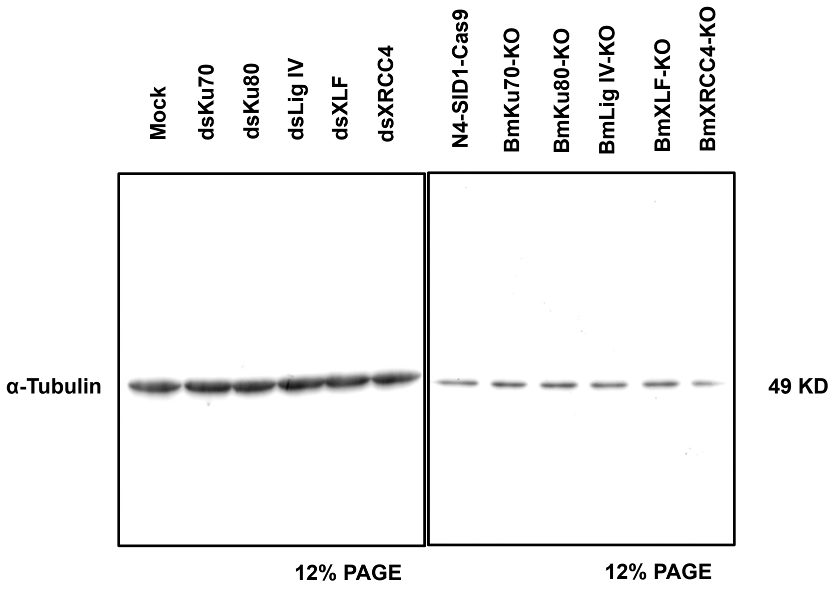

Fig. 4b

Fig. 4c

Supplementary Table S1: Primers used for vector construction

Primer name	Primer sequence (5'-3')	Use
Hcas9-withoutATG-F	GATAAAAAGTATTCTATTGGTTTAGaCATCGGCA	cloning of Cas9 CDS
Hcas9-NotI-R	TTGCGGCCGCAATCATCCTGCAGCCTTGTCATCGTC	
Hcas9-R-D10A	AGTGCCGATgcgTAAACCAATAGAATACTTTTTATCCAT	Cas9 D10A mutation
Hcas9-F-D10A	AATTCCGTTGGATGGGCTGTCATAACCGATGAATACAAA	
Hcas9-R-H840A	TTGGGGTACAATggcATCGACGTCGTAATC	Cas9 H840A mutation
Hcas9-F-H840A	TCCTTTTTGAAGGACGATTCAATCGACAATAAAG	
BmU6-2 promoter-F	AGGTTATGTAGTACACATTG	cloning of U6 promoter
BmU6-2 promoter-R	CACTTGTAGAGCACGATATT	
chiRNA-R-polyT	AAAAAAgcaccgactcggtgccactttttca	cloning of U6-gRNA cassette
NHEJ I-SceI mCherry atg	ACGCTAGGGATAACAGGGTAATCCACCATGGTGAGCAAGGGCGAGGAGGATAAC	NHEJ reporter construction
NHEJ I-SceI IE2 polyA	CTATATTACCCTGTTATCCCTAGCGTAACCGTATTACCGCCTTTGAGTGAGC	
NHEJ I-SceI linker a	AGCTTTACGCTAGGGATAACAGGGTAATATACC	
NHEJ I-SceI linker b	AGCTGGTATATTACCCTGTTATCCCTAGCGTAA	

Supplementary Table S2: Primers used for gRNA cloning into All-In-One or psk-U6-gRNA backbone

Gene	Primer name/gRNA/Target		Sequence (5' to 3')
BmKU70	Ku70-gRNA-F		GCTCAGCTTATTTTTGAAATgttttagagctagaaatagcaagtt
	gRNA		GGCTCAGCTTATTTTTGAAAT
	Target		CGCTCAGCTTATTTTTGAAATTGG
BmKU80	Ku80-gRNA-F		AAATCAGATTCAAGAACGAAgttttagagctagaaatagcaagtt
	gRNA		GAAATCAGATTCAAGAACGAA
	Target		AAAATCAGATTCAAGAACGAAAGG
BmLIG IV	Lig IV-gRNA-F		CATGTGTAGCTATTTTATCAgttttagagctagaaatagcaagtt
	gRNA		GCATGTGTAGCTATTTTATCA
	Target		CCTTGATAAAATAGCTACACATGC
BmXRCC4	XRCC4-gRNA-F		AGAGTATACTGATGATTCTGgttttagagctagaaatagcaagtt
	gRNA		GAGAGTATACTGATGATTCTG
	Target		AAGAGTATACTGATGATTCTGAGG
BmXLF	XLF-gRNA-F		AATATTAACTGTCGATAATGgttttagagctagaaatagcaagtt
	gRNA		GAATATTAACTGTCGATAATG
	Target		AAATATTAACTGTCGATAATGTGG
BmTUDOR-SN	Targeting to site 1	SN2-gRNA-F	GAAAGACGTCGAAGTAGTTCgttttagagctagaaatagcaagtt
		gRNA	GGAAAGACGTCGAAGTAGTTC
		Target	AAGGACGATGCAGTGGAATTCTGG
	Targeting to site 2	Tudor-gRNA-F	GTTTTACAGCCGATGACCAAgttttagagctagaaatagcaagtt
		gRNA	GGTTTTACAGCCGATGACCAA
		Target	CGTTTTACAGCCGATGACCAATGG
	Targeting to site 3	Tend-gRNA-F	AGGACGATGCAGTGGAATTCgttttagagctagaaatagcaagtt
		gRNA	GAGGACGATGCAGTGGAATTC
		Target	GAGGACGATGCAGTGGAATTCGGG
	For double cut	Tend-D10A-gRNA1	AATTCCACTGCATCGTCCTgttttagagctagaaatagcaagtt
		gRNA	GAATTCCACTGCATCGTCCT
		Target	CCGAGGACGATGCAGTGGAATTC
	For double cut	Tend-D10A-gRNA2	CGTCGCTAGCTGTACGCCCCgttttagagctagaaatagcaagtt
		gRNA	GCGTCGCTAGCTGTACGCCCC
		Target	GCGTCGCTAGCTGTACGCCCCCGG

The lower case letters in the primers indicate the sequence in the backbone of All-In-One and psk-U6-gRNA plasmids. The red marked nucleotides indicate the perfect marched regions between gRNAs and targets in genome. PAMs were marked by blue.

Supplementary Table S3: Primers used for donor construction

Donor name	Primer name	Sequence (5' to 3')
Donor 1	S-HR-uparm-F	ATTTGGAGTACTGATGCTGATGTATGTTACG
	S-HR-uparm-R	CTTTCTGCAGCAATCTGGACTCCAGGAAG
	S-HR-downarm-F	GACGTCGAAGTAGTTCTGGAGTCCGTT
	S-HR-downarm-R	GGCTTTCAATGTAGATGCACCTGTATTTCAT
Donor 2	T-HR-uparm-F	GTCGTAAGCGGTGGCACGTAAG
	T-HR-uparm-R	TGGTCATCGGCTGTAAAACGAGC
	T-HR-downarm-F	ATGGTACAGGGCAAAGATTGAAAAAATAAC
	T-HR-downarm-R	CAATCTGGTTATGTCTAACGTCTGAAAATATATTA
Donor 4	T-end-uparm-F	GTTAGACATAACCAGATTGGCAGCACT
	T-end-uparm-R	GCGACGCACCCCGAATTCTAC
	T-end-downarm-F	GCTGTACGCCCCCGGCGTTG
	T-end-downarm-R	TACCAAGTTCTTTAACAATCCACGCACCTA
L-I-SceI	plits-A036-ISceI-R2	TGTTATCCCTATGGCGTAATCATGGTCATAGCTGTTTCC
	plits-upT7-ISceI-F2	GGGTAATAGCTACGTAATACGACTCACTATAGG
R-I-SceI	plits-downT7-ISceI-R2	TGTTATCCCTACGTAATACGACTCACTATAGG
	plits-A001-ISceI-F2	GGGTAATGACTGGCCGTCGTTTTACAACGTCGT
LR-I-SceI	plits-A036-ISceI-R2	TGTTATCCCTATGGCGTAATCATGGTCATAGCTGTTTCC
	plits-upT7-ISceI-F2	GGGTAATAGCTACGTAATACGACTCACTATAGG
	plits-downT7-ISceI-R2	TGTTATCCCTACGTAATACGACTCACTATAGG
	plits-A001-ISceI-F2	GGGTAATGACTGGCCGTCGTTTTACAACGTCGT
Vector donor 500 bp arm	T4-Donor500bp-F	TCTTATATTAATCCTGTTAATTTTTGTTTCGGCTTAC
	T4-Donor500bp-R	ACACATTTATATTATGACCAACAACAGGCAT
Vector donor 250 bp arm	T4-Donor250bp-F	TACAAGCCGAAACGCATTACTGCTT
	T4-Donor250bp-R	TTCACTGCTCACCATCGTAATGTCC
Vector donor 100 bp arm	T4-Donor100bp-F	CATTGATGGCCGAGTATCGCGC
	T4-Donor100bp-R	TGGTAAAGAATGGCGTGGACGAT
Vector donor 25 bp arm	T4-Donor25bp-F	TGCAGTAGAATTCGGGGTGCGTCGC
	T4-Donor25bp-R	GGCGTCAACGCCGGGGGCGTACAGC
For all donors	EGFP-full-F	ATGGTGAGCAAGGGCGAGGA
	EGFP-full-R-outstop	CTTGTACAGCTCGTCCATGCCG
	pZERO2-EcorV-R	ATCTGCAGAATTCCAGCACACTGG
	pZERO2-EcorV-F	ATCCATCACACTGGCGGCC

The red marked T in the primer "T-end-uparm-R" was designed to avoid gRNA attaching after genome editing.

Supplementary Table S4: Primers used for dsRNA synthesis and RT-PCR

Gene	Primer name	Sequence (5' to 3')
BmKU70	Ku70-N4dsRNA1-F	TAGCGACCGCTCAGCTTATT
	Ku70-N4dsRNA1-R	GCTGGACCAAATGGGTTATTT
BmKU80	Ku80-N4dsRNA1-F	TTTATGATATGGCGCGTGAA
	Ku80-N4dsRNA1-R	AGCCTTGAAACCATTCAGGA
BmLIG IV	Lig IV-N4dsRNA1-F	ACTCATCTGCCTCCTGCACT
	Lig IV-N4dsRNA1-F	ATGGTAGCATTTCACTCGGG
BmXRCC4	XRCC4-N4dsRNA1-F	TGGAGATGGAATCCTGAAGAA
	XRCC4-N4dsRNA1-R	GGTTGTGGGTACATCATCCC
BmXLF	XLF-N4dsRNA1-F	AACCTACTGAAGATAATCAATGATCTGT
	XLF-N4dsRNA1-R	GATCGACTTTTGTAGTTTCGCGT
BmGAPDH	GAPDH-RT5	GGCCGCATTGGCCGTTTGGTGCTCCG
	GAPDH-RT3	GTGGGGCAAGACAGTTTGTGGTGCAAGAAG

Supplementary Table S5: Primers used for Genomic DNA PCR

Purpose	Primer name	Sequence (5' to 3')	Length
Ku70 gRNA targeting site	Ku70-site-F	GAAGTAGAAGAGTGCGAAGAGTTTTC	861 bp
	Ku70-site-R	GTGAAGCAGCTCAGACTTAAGTAAAGGC	
Ku80 gRNA targeting site	Ku80-site-F	GTTGCTGAGCCTTTTGGATCGGC	845 bp
	Ku80-site-R	GGATTCAGACTTCGAAATGCAATACAGTCT	
Lig IV gRNA targeting site	Lig IV-site-F	TCCCTTGGTTCATTGCTGGTG	970 bp
	Lig IV-site-R	ACTAACAATCACGCCACGTTAACTG	
XLF gRNA targeting site	XLF-site-F	CAATGTCATAAAATTAGTGTGAACGTGAACTG	752 bp
	XLF-site-R	TTAAAATGTAGACTTTCCAGAGTATCAGATGAAATG	
XRCC4 gRNA targeting site	XRCC4-site-F	CTTTGGAGATGGAATCCTGAAGAAC	569 bp
	XRCC4-site-R	GGGAGTATCAAATGTTTATAATTCAGAAAG	
D1 5' junction	D1-5-F	CCAAAGATGAAAATGTTACTGAAGCCTTG	1574 bp
	EGFP-R	GTTGTACTCCAGCTTGTGCCCCAGGATG	
D2 5' junction	D2-5-F	ATGTACGAGGCACGTGAATTTCTTAG	1555 bp
	EGFP-R	GTTGTACTCCAGCTTGTGCCCCAGGATG	
D3 5' junction	D1-5-F	CCAAAGATGAAAATGTTACTGAAGCCTTG	1574 bp
	EGFP-R	GTTGTACTCCAGCTTGTGCCCCAGGATG	
D4 5' junction	D4-5-F	CGCTGCGAACTATACCACTACCTTT	1336 bp
	EGFP-R	GTTGTACTCCAGCTTGTGCCCCAGGATG	
D1 3' junction	EGFP-F	TACCCCGACCACATGAAGCAGCACGAC	1678 bp
	D1-3-R	ATGTGTTTTAACAACGAGCGCGTCT	
D2 3' junction	EGFP-F	TACCCCGACCACATGAAGCAGCACGAC	1574 bp
	D2-3-R	CCTCTTGCCAACGACATCATTATAGAA	
D3 3' junction	EGFP-F	TACCCCGACCACATGAAGCAGCACGAC	1574 bp
	D2-3-R	CCTCTTGCCAACGACATCATTATAGAA	
D4 3' junction	EGFP-F	TACCCCGACCACATGAAGCAGCACGAC	1557 bp
	D4-3-R	TGTATTTATAATCTGCGAATACAAACAAAAAACTTTGT	
Loading control	Reference-F (D2-5-F)	ATGTACGAGGCACGTGAATTTCTTAG	2211 bp
	Reference-R (D2-3-R)	CCTCTTGCCAACGACATCATTATAGAA	
